# Triboelectric Wearable Sensors for Human-Centric Smart Electronics: From Self-Powered Sensing to Artificial Intelligence-Assisted Human–Machine Interface Systems

**DOI:** 10.1007/s40820-026-02263-z

**Published:** 2026-06-16

**Authors:** Yoonsang Ra, Sumin Cho, Xinge Guo, Dongwhi Choi, Chengkuo Lee

**Affiliations:** 1 Department of Mechanical Engineering (Integrated Engineering Program), Kyung Hee University, 1732 Deogyeong-daero, Yongin, Gyeonggi 17104 Republic of Korea; 2 School of Mechanical Engineering, Chonnam National University, 77 Yongbong-ro, Buk-gu, Gwangju, 61186 Republic of Korea; 3 Department of Bioengineering, University of California, Los Angeles, CA USA; 4 Department of Electrical & Computer Engineering, National University of Singapore, 4 Engineering Drive 3, Singapore, 117576 Singapore; 5 Center for Intelligent Sensors and MEMS (CISM), National University of Singapore, 5 Engineering Drive 1, Singapore, 117608 Singapore; 6 NUS Graduate School - Integrative Sciences and Engineering Program (ISEP), National University of Singapore, Singapore, 119077 Singapore; 7 Research Center for Sustainable Urban Farming (SUrF), National University of Singapore, Singapore, 117558 Singapore

**Keywords:** Human–machine interface, Smart electronics, Wearable sensor, Triboelectric nanogenerator, Artificial intelligence

## Abstract

As intelligent electronics become increasingly integrated into daily life, health care, virtual interaction, and assistive systems, human–machine interfaces (HMIs) require sensing platforms that are not only wearable and self-powered but also capable of translating human signals into adaptive machine functions. Triboelectric wearable sensors are particularly attractive in this regard because they directly transduce human-generated mechanical stimuli, provide broad material and structural design freedom, and are readily adaptable to body-interfaced formats. In this review, wearability refers to body-mounted, skin-interfaced, textile-integrated, or otherwise human-attached triboelectric sensing platforms, whereas human-centric smart electronics refers to downstream electronic systems that remain functionally anchored to human-originated sensing, interpretation, feedback, or control. From this perspective, we review triboelectric wearable sensors from fundamentals to applications, covering working principles, material selection, device architectures, and fabrication strategies. We further discuss artificial intelligence-assisted signal processing, triboelectric artificial synapses, and neuromorphic computing as key bridges from self-powered sensing to intelligent HMI. Representative application spaces, including health care, gesture recognition, device control, immersive virtual interaction, wearable-to-robotic extensions, and intelligent transportation are discussed only when wearable triboelectric sensing serves as the primary human–input interface. Finally, the remaining challenges and future directions toward next-generation human-centric smart electronics are outlined.

## Highlights

- A wearable-centered review framework is presented for triboelectric sensors, covering self-powered sensing principles, material selection, device architectures, and fabrication strategies.
- Artificial intelligence-assisted signal processing, triboelectric artificial synapses, and neuromorphic computing are identified as key bridges from self-powered sensing to adaptive human–machine interfaces.
- Representative applications and future directions are organized toward human-centric smart electronics, including health care, gesture interaction, robotics, intelligent transportation, and next-generation embodied systems.

## Graphical abstract

**Figure:**
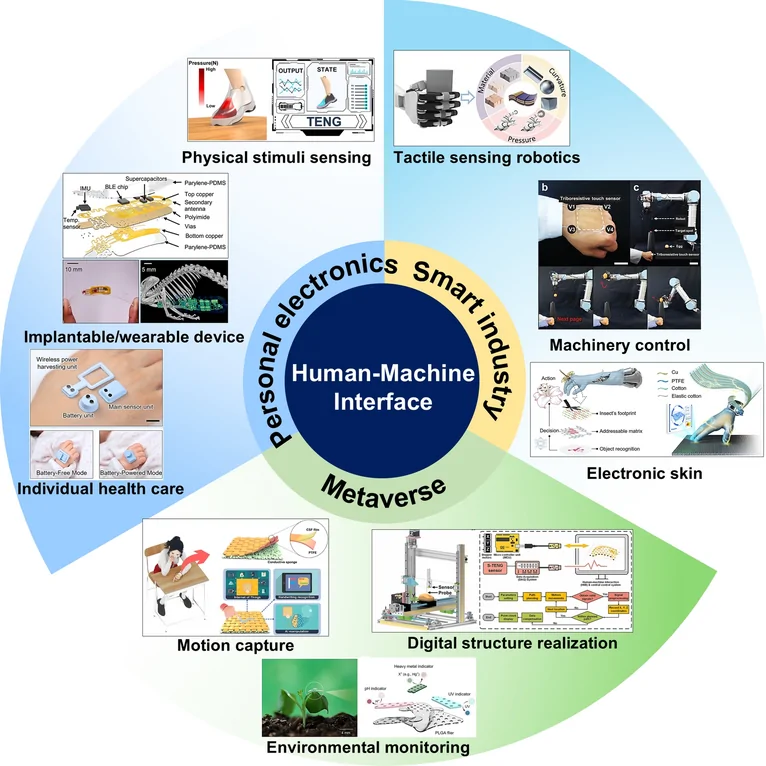

## Introduction

**Fig. 1:**
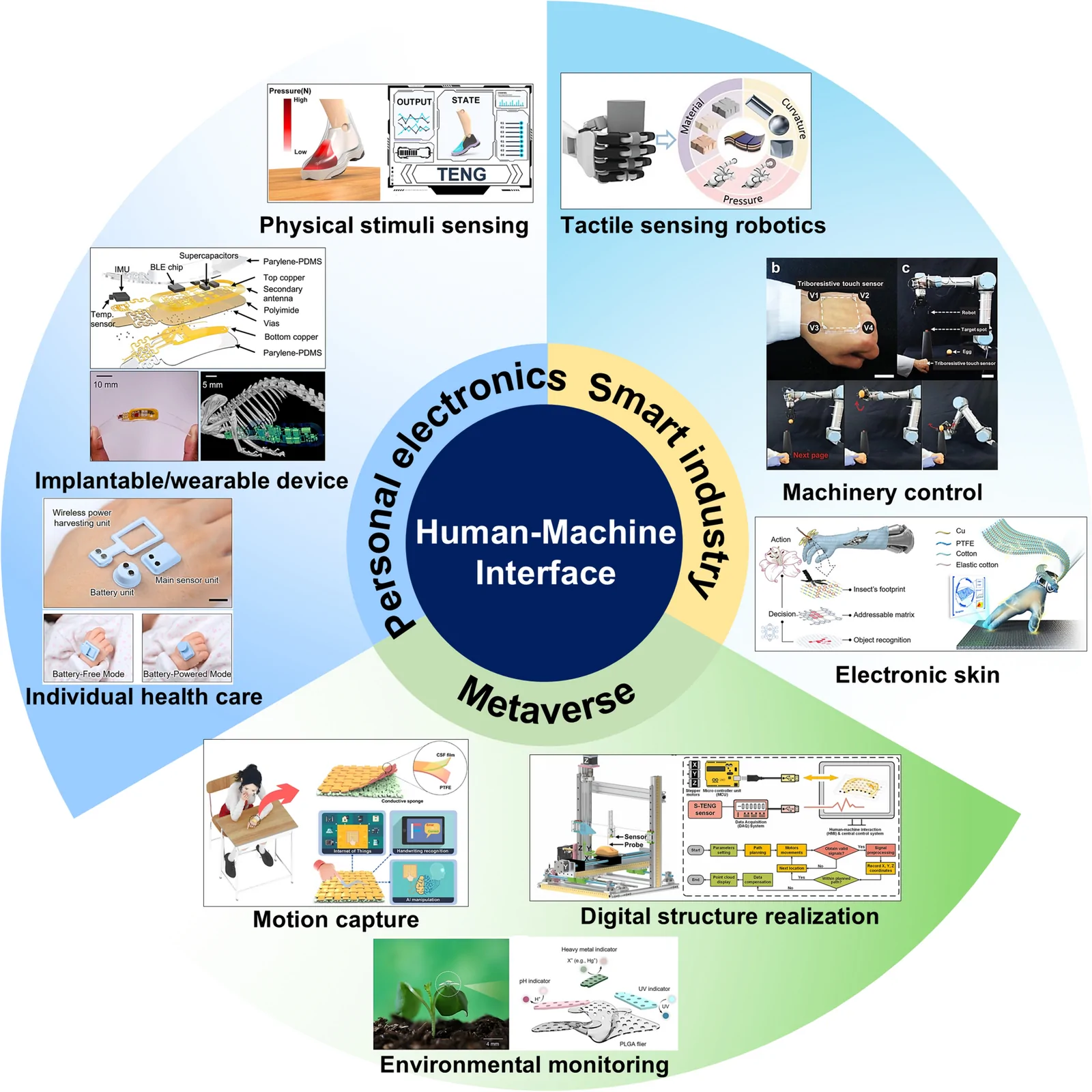
HMI technologies playing varied and crucial roles in next-generation human–machine interactions for the development of the automated and intelligent future lifestyle: Reprinted with permission from Ref. [5]. © 2023 Elsevier B.V. All rights reserved. Reprinted with permission from Ref. [6]. © 2024 Elsevier Inc. Reprinted with permission from Ref. [7]. Copyright © 2022, American Chemical Society. Reprinted with permission from Ref. [8]. © 2022 Wiley‐VCH GmbH. Reprinted with permission from Ref. [9]. © 2023 Elsevier Inc. Reprinted with permission from Ref. [10]. © 2024 Wiley‐VCH GmbH. Reprinted with permission from Ref. [11]. Copyright © 2022, Shen, S., et al. Reprinted with permission from Ref. [12]. Copyright © 2022, The American Association for the Advancement of Science. Reprinted with permission from Ref. [13]. © 2022 Elsevier Ltd. All rights reserved

The rapid development of artificial intelligence (AI), the Internet of Things (IoT), and data-driven automation is accelerating the transition from conventional electronics to interactive intelligent systems [1]. As these systems become more deeply embedded in health care, assistive technologies, smart manufacturing, and virtual environments, their performance increasingly depends not only on device-level capability but also on how effectively they acquire, interpret, and respond to human signals. In this regard, human–machine interface (HMI) technologies have become a central component of next-generation smart electronics, because they define the quality of communication between humans and intelligent machines [2–4]. Accordingly, future electronics are moving toward human-centric smart electronics for the development of intelligent electronics [5–7], smart industries [8–10], and the metaverse [11–13] around human physiology, motion, intention, comfort, and personalization, as presented in Fig. 1.

Among the many components of HMI systems, sensors are the primary front-end elements that capture human and environmental information, including body motion, physiological activity, and device interaction states [14–18]. In particular, self-powered wearable sensors have attracted considerable attention because they can directly detect human-originated stimuli while reducing dependence on external power sources and improving flexibility, lightweight integration, and user comfort [19–29]. Among self-powered sensing technologies, triboelectric sensors are especially promising owing to their simple working mechanism, wide material availability, structural versatility, and compatibility with flexible, stretchable, textile-based, and skin-interfaced devices [30, 31]. Many researchers have reported that the self-powered wearable sensor can be applied to a wide range of fields such as individual health care [32], human motion capture [33], environmental monitoring [34], robotics with tactile sensing [35], and electronic skin [36]. Self-powered sensors are particularly attractive for HMI because they can directly convert external stimuli into electrical output without relying on external power supplies or batteries. Compared with conventional sensors, this enables lower power dependence, simpler system integration, improved wearability, and more continuous operation in human-centered interactive electronics. Among the various self-powered sensing technologies, triboelectric sensors are especially suitable for wearable HMI because they combine simple operating principles, broad material choices, and strong design flexibility with compatibility for flexible, stretchable, and skin-interfaced formats. These features have enabled a wide range of representative wearable triboelectric devices, while also highlighting remaining challenges in stability, signal interpretation, and practical system integration. Recently, efforts to develop AI-assisted wearable sensor technologies for the higher level of HMI have been highlighted with the advent of the Artificial Intelligence of Things (AIoT), which is a concept of the combination of AI and IoT [37]. With AI technology, the self-powered wearable sensor has set the stage for a leap to the next level beyond just detection of stimuli [38]. The introduction of AI does not directly alter the intrinsic physical sensitivity of the sensor, which is primarily determined by material selection, device structure, and operating mechanism. Instead, AI enhances the interpretation and utilization of triboelectric sensing signals by improving noise filtering, feature extraction, pattern recognition, multimodal data fusion, and user-specific adaptation, thereby increasing the overall accuracy, reliability, and practical functionality of wearable sensing systems [39]. Furthermore, the integration of AI has pushed triboelectric wearable sensors beyond simple stimulus detection, enabling higher-level functions such as feature extraction, pattern recognition, multimodal fusion, adaptive learning, and personalized interaction.

In this review, wearability refers to triboelectric sensing platforms that are body-mounted, skin-interfaced, textile-integrated, or otherwise directly attached to the human body for acquiring human-generated mechanical or physiological signals [40, 41]. Meanwhile, human-centric smart electronics refers to electronic systems that remain functionally centered on these human-originated inputs for sensing, interpretation, feedback, and control [42]. From this viewpoint, the scope of this review is intentionally broader than body-worn device hardware alone, but narrower than general triboelectric systems [43–47]. In this regard, many novel applications with high-end performance for seamless human–machine interaction have been proposed based on the triboelectric wearable sensor combined with AI technology [48–51]. Specifically, we include artificial synapses and neuromorphic computing because they serve as intelligence processing bridges that convert self-powered triboelectric sensing into adaptive HMI. We also include downstream examples in device control, virtual interaction, robotics, transportation, and potentially future applications only when wearable triboelectric sensors act as the primary human–input interface or directly enable human-centered interactive functions [52].

Based on this framework, this review focuses on the system-level transition from self-powered triboelectric wearable sensing to AI-assisted human-centric HMI systems [53]. We discuss the fundamental principles, material characteristics, and fabrication strategies of triboelectric sensors. We then examine AI-enabled signal interpretation, triboelectric artificial synapses, and neuromorphic computing as enabling routes toward intelligent sensing. Finally, we summarize representative human-centered application strategies and discuss the remaining challenges and future directions for next-generation smart electronics [54–56].

## Triboelectric Materials

With attracting a lot of attention, in-depth research on the principle, influential factors, and applications of triboelectric sensors have been conducted. As with other self-powered sensors, the components and materials of the triboelectric sensor are one of its most important parts. Many researchers emphasize the importance of materials as a major factor in the triboelectric sensor for improving its performance and functionality. In this section, we discuss the major materials of the triboelectric sensor in terms of the fundamental principle and essential components.

### Fundamental Principle and Working Mechanism

**Fig. 2:**
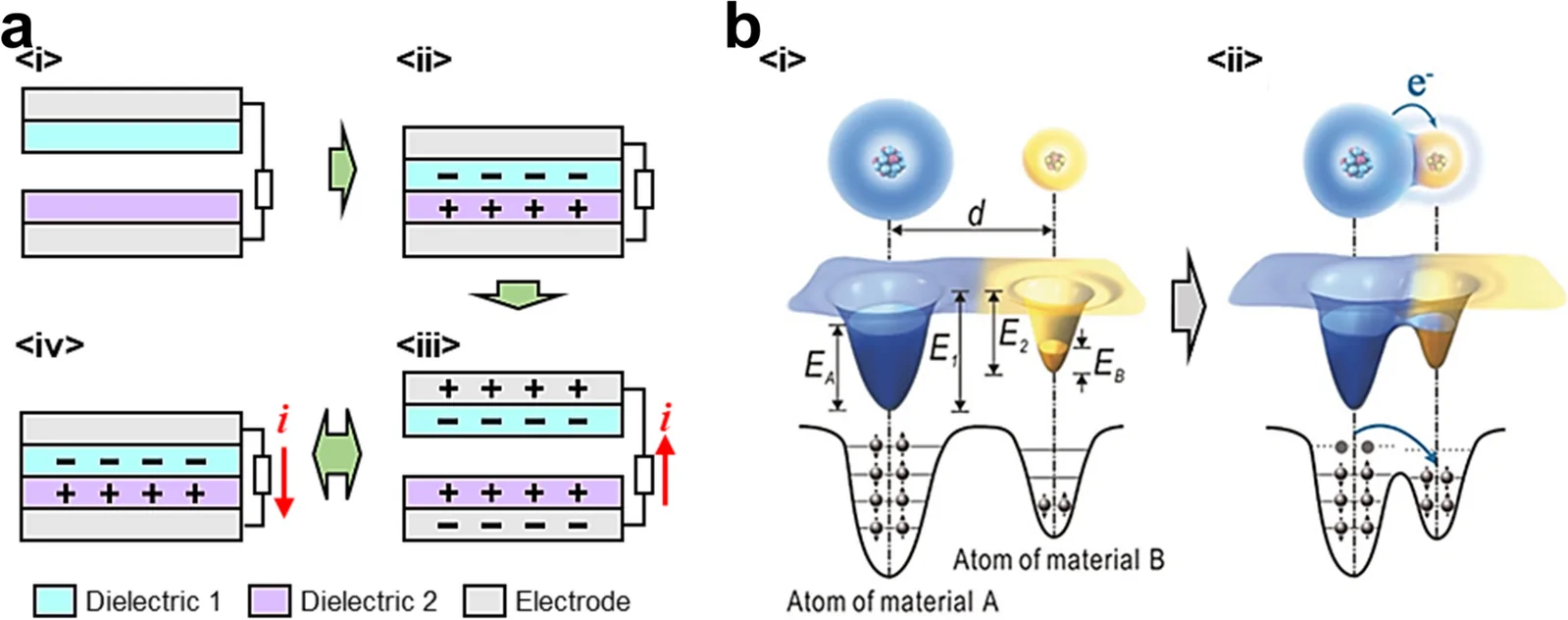
**a** Working mechanism of triboelectric sensor and **b** fundamental principle of triboelectric effect. Reprinted with permission from Ref. [57]. © 2018 WILEY‐VCH Verlag GmbH & Co. KGaA, Weinheim

As mentioned above, the triboelectric sensor operates based on the working principle of TENG. It generates electricity by the sequential contact and separation of two different material surfaces. Figure 2a shows the working mechanism and the movement of charges when the triboelectric sensor is working with the vertical contact/separation mode as an example. In the operation of triboelectric sensors, triboelectric effect in the dielectric contact surfaces and charge induction phenomenon in adjacent conducting materials occur. When the surfaces of two different materials come into contact, electrical charge pairs are generated in the interface by the triboelectric effect between the materials as shown in Fig. 2a<i> and <ii>. Then, with the separation of the two surfaces, the charge pairs are also physically separated and remained charges on both surfaces become the net charges. The net charges generate local electric fields and lead induction of opposite charges in the nearby conducting materials. This opposite charge induction makes an electric current in the connected wire as shown in Fig. 2a<iii>. When the two dielectric surfaces come into contact with each other again, the net charges on the surfaces physically form the charge pairs again and the induced charges in the electrodes generate an opposite current to the previous current direction in the separation stage as shown in Fig. 2a<iv>. Therefore, the movement of the inductive charges shows the peak-type alternating current at the exact timing of the contact and separation of the two surfaces.

Figure 2b shows the charge transfer phenomenon with an electron cloud model to describe the occurrence of the triboelectric effect when two different material surfaces come into contact with each other [57]. As shown in Fig. 2b<i>, the electrons of atoms and molecules form the electron clouds. The electron cloud includes the atomic or molecular orbitals and each atom is assumed as a potential well containing electrons. The distance between the electron clouds of the atoms of two materials, A and B, is defined as *d*. *E*_*1*_ and *E*_*2*_ are the depths of potential wells, which are the required energies for electrons to escape from the surfaces of materials A and B, respectively. *E*_*A*_ and *E*_*B*_ indicate the occupied energy levels of electrons in the atoms of materials A and B, respectively. In the normal condition, the electrons are confined in the potential well because *E*_*A*_ and *E*_*B*_ are smaller than *E*_*1*_ and *E*_*2*_, respectively. When the surfaces of the materials A and B come into contact with each other, the electron clouds are overlapped and thus, *E*_*1*_ and *E*_*2*_ decrease. Here, if *E*_*1*_ becomes smaller than *E*_*A*_, the electrons of the atom of material A that have bigger energy than the decreased *E*_*1*_ can move to the atom of material B as shown in Fig. 2b<ii>. Then, after the materials A and B are separated from each other, *E*_*1*_ and *E*_*2*_ are restored to their original level and the moved electrons are kept in the potential well of the atom of material B. As a result, the materials A and B remain in positively and negatively charged states, respectively.

### Major Components and Materials

**Fig. 3:**
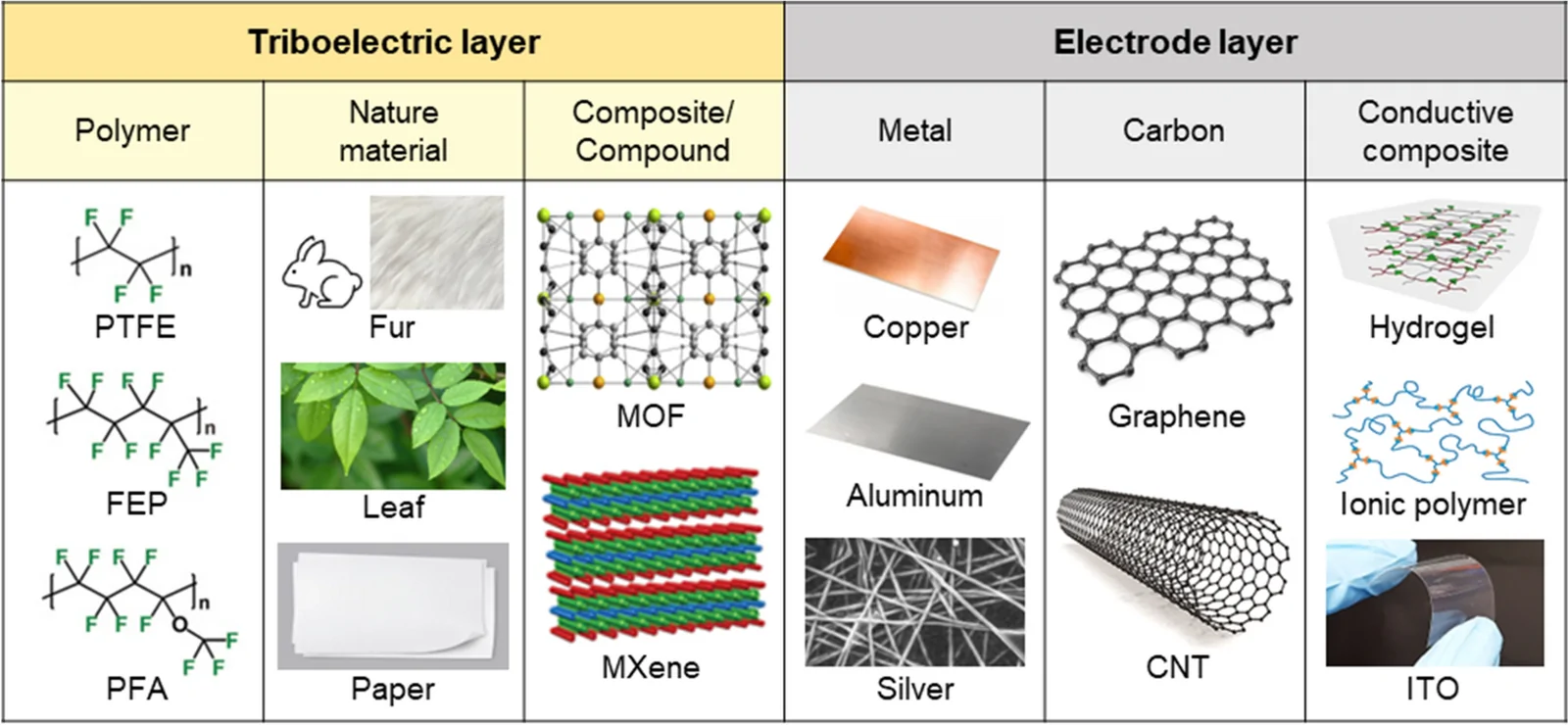
Major components and materials of the triboelectric sensor. Reprinted with permission from Ref. [71]. Copyright © 2023 Elsevier Ltd. All rights reserved. Reprinted with permission from Ref. [72]. © 2020 Elsevier Ltd. All rights reserved. Reprinted with permission from Ref. [73]. © 2024 Elsevier Ltd. All rights are reserved, including those for text and data mining, AI training, and similar technologies. Reprinted with permission from Ref. [74]. © 2021 Wiley‐VCH GmbH

Based on the working mechanism and fundamental principle, the triboelectric sensor needs two types of layers, the triboelectric and electrode layers, as the essential components for its operation. The triboelectric layer is made of dielectric material for constructing the net charges with the triboelectric effect. Therefore, the polymer is one of the most widely employed materials for the triboelectric layer. In particular, fluorine-containing polymers such as polytetrafluoroethylene (PTFE), fluoroethylene propylene (FEP), and perfluoro alkoxy alkane (PFA) have been verified as one of the most useful materials for the triboelectric layer because their fluorinated molecular structures exhibit a strong tendency to gain and retain electrons during contact electrification [58]. Moreover, the polymers can be optimized to the desirable condition for the application with their advantageous mechanical and chemical properties including durability, chemical resistance, cost-effectiveness, easy processing, corrosion resistance, and thermostability. Nature materials have also been widely adopted such as animal fur, leaf, and paper. The animal fur has good durability and high sensitivity to external stimuli [59]. The leaf from a plant has micro- and nanoscale surface structures that can achieve a localized contact pressure and large surface area [60]. Paper made from wood pulp is one of the most popular natural materials with its advantages including economic feasibility, biodegradability, and recyclability [61]. Furthermore, many researchers have been trying to develop nature-inspired materials mimicking and achieving the superior characteristics of natural materials such as surface structure, self-healing, and biocompatibility [62]. Recently, the composites and compounds have emerged as the promising materials for the triboelectric layer. The triboelectric sensors based on the metal–organic framework (MOF), which is a class of crystalline porous solids composed of a three-dimensional network of metal ions held in place by multidentate organic molecules, have actively developed with its extraordinary design ability and functionality [63]. The triboelectric effect can also occur on liquid surfaces not only the solid materials. The triboelectric effect on liquid surfaces enables droplet manipulation, particle arrangement, energy harvesting, and sensing [64]. In this respect, the triboelectric sensors based on the liquid–solid contact have been actively researched [65]. When the triboelectric effect occurs on the triboelectric layer, the opposite charges have to be induced on the electrode layer as explained in the working mechanism section so that the induced movement of charges can be measured as a current. Therefore, the electrode layer is made of highly conductive materials such as metal, carbon, and conductive composites. Copper, aluminum, and silver are widely used thanks to their availability, cost-effectiveness, and good conductivity [66]. Carbon-based materials such as graphene, carbon nanotube (CNT), and carbon fiber have also been attracting attention as a beneficial member of the triboelectric sensor with their advantageous mechanical and electrical properties including robustness, flexibility, lightweight, and high-level electrical conductivity [67]. Indium tin oxide (ITO) is also one of the most popularly adopted materials for triboelectric sensors because it is transparent, flexible, and conductive [68]. Conductive hydrogels and ionic polymers have emerged recently as the most highlighted materials for triboelectric wearable sensors with their flexibility, stretchability, eco-friendliness, skin attachability, and biocompatibility [69, 70]. The major components and described materials are summarized in Fig. 3 [71–74].

### Triboelectric Series

**Fig. 4:**
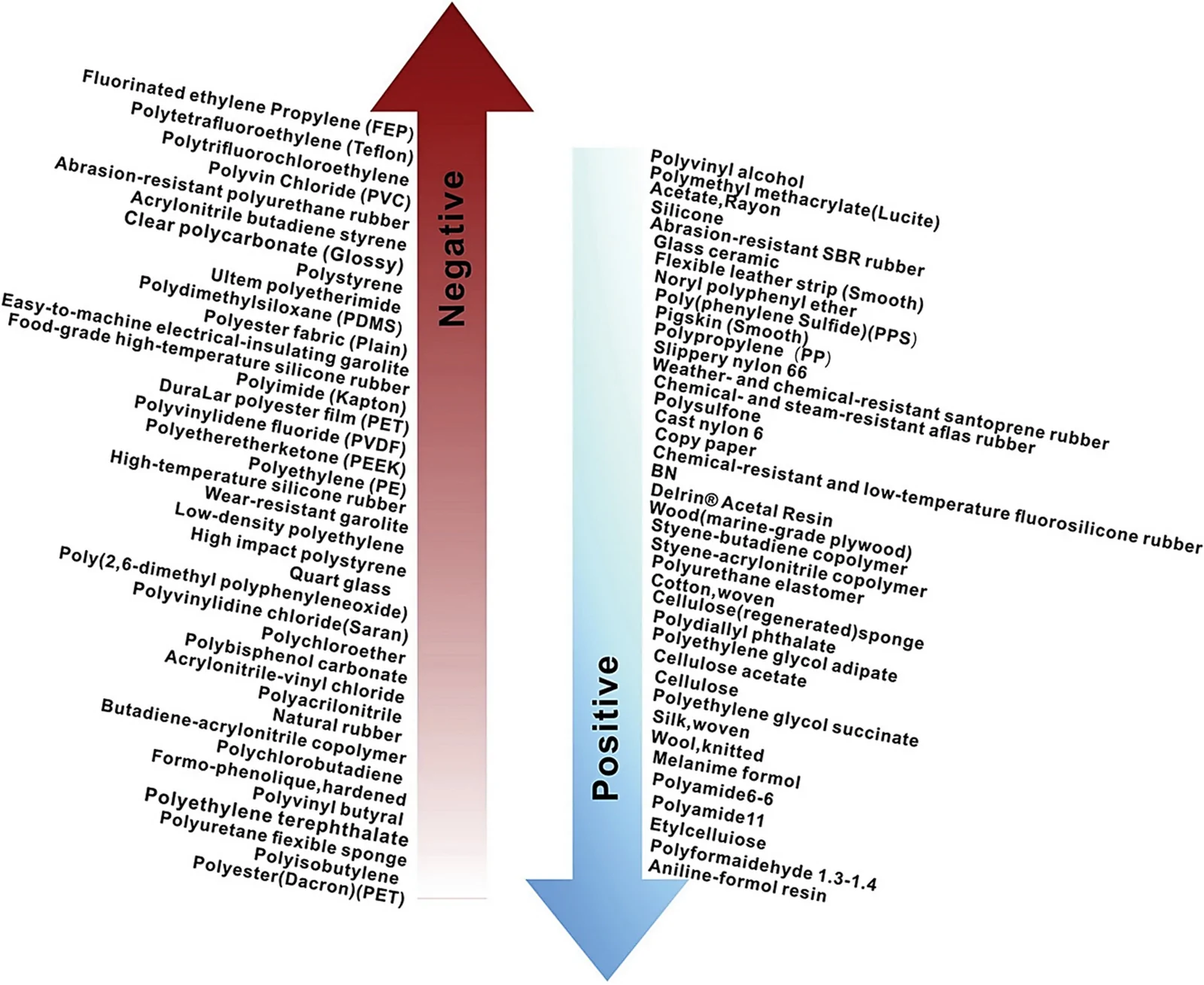
Triboelectric series. Reprinted with permission from Ref. [82]. © 2020 Wiley‐VCH GmbH

Although both the triboelectric and electrode layers are necessary to the triboelectric sensor, the major factor that affects the performance of the triboelectric sensor has been considered the electrical characteristic of the material of the triboelectric layer [75–78]. The triboelectric sensor performance is mainly determined by the generated charge density on the contact surface based on its working mechanism. Moreover, the electrons have to be able to transfer easily from one potential well to another of the contact materials based on the fundamental principle of the triboelectric effect so that the amount of the generated charge pairs increases. Given that, the triboelectric characteristics of the various materials have been analyzed with wide attention and a number of research groups have reported triboelectric series [79–81]. The triboelectric series is a list that ranks materials depending on their tendency to gain or lose electrons as shown in Fig. 4 [82]. It indicates how many a material makes positive or negative charges relative to others on the list. Therefore, we can get the optimal pair of triboelectric materials, which consists of one being positively well charged and the other being negatively well charged when they come into contact, for the target performance and potential application. For example, FEP, PTFE, polyvinyl chloride (PVC), polydimethylsiloxane (PDMS), and polyimide (PI, Kapton) are the representative materials for good negative triboelectric materials. Polyamide 6–6 (nylon) and human skin are considered good positive triboelectric materials.

## Self-Powered Triboelectric Sensors for Wearable HMIs

**Fig. 5:**
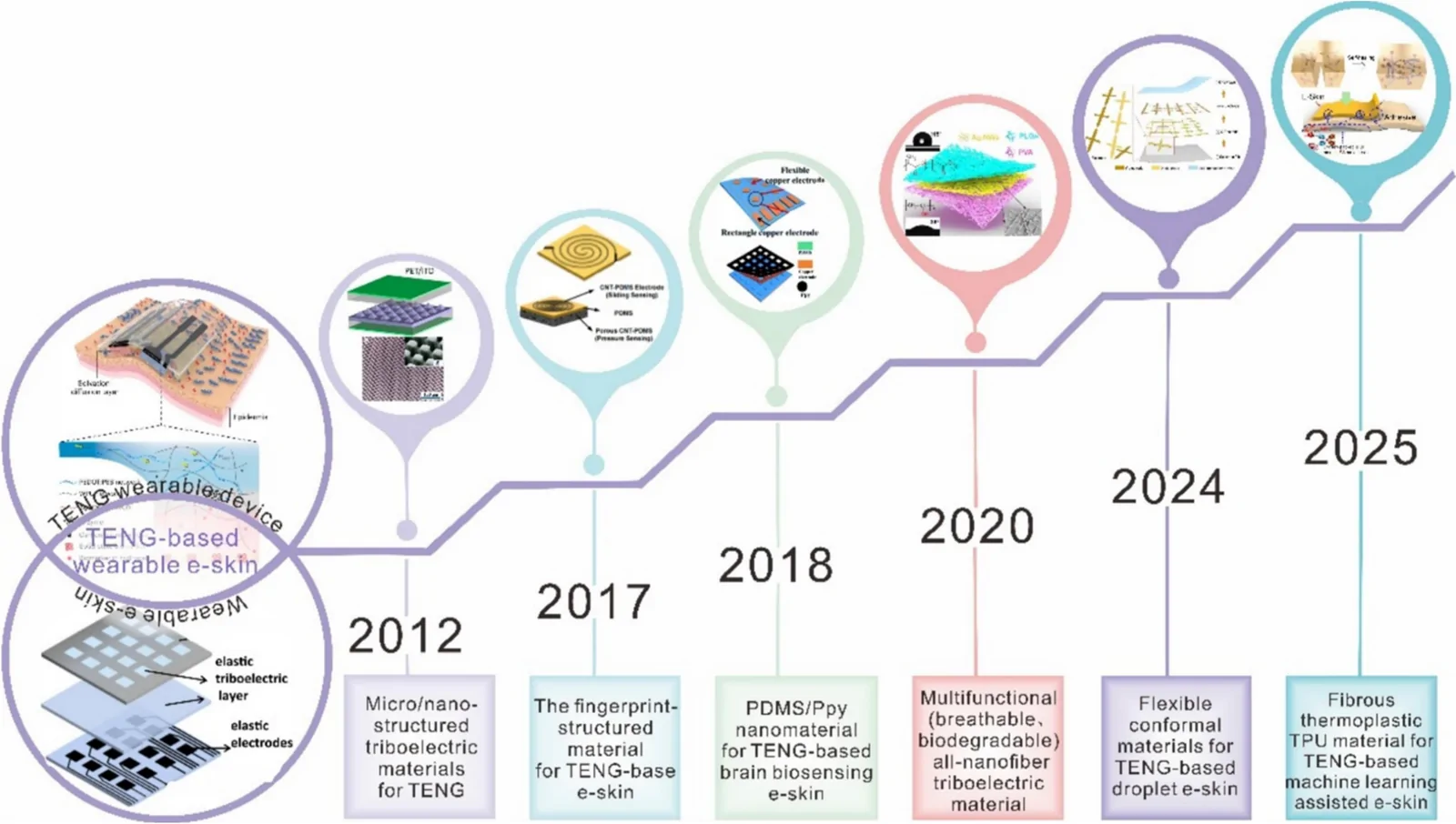
Rapid development of self-powered wearable sensor technologies. Reprinted with permission from Ref. [88]. © 2025 Elsevier Ltd. All rights are reserved, including those for text and data mining, AI training, and similar technologies

As the personalization and optimization of electronics are increasingly emphasized, direct sensing of user-originated information such as motion, voice, heartbeat, and gait has become highly important [83–85]. In this regard, wearable sensors have emerged as a promising platform, and self-powered sensing technologies have attracted broad interest because they can convert external stimuli into electrical output without relying on conventional batteries or grid-based power sources [33, 86, 87]. Among them, triboelectric sensors have shown particularly rapid progress in wearable and skin-interfaced systems because of their broad material choices, structural design versatility, and compatibility with multifunctional integration. Figure 5 summarizes the recent development trend of triboelectric wearable sensing materials and device platforms [88]. In contrast to earlier development stages centered mainly on microstructured triboelectric interfaces, recent progress has expanded toward intelligent wearable e-skin systems with greater multifunctionality and environmental adaptability. Representative directions include the progression from patterned PDMS-based triboelectric materials to fingerprint-structured e-skin, triboelectric–electrochemical hybrid materials, breathable biodegradable all-nanofiber materials, flexible conformal droplet e-skin, and machine learning-assisted fibrous thermoplastic e-skin. Furthermore, recent triboelectric wearable systems are increasingly being organized through 1D fibers, 2D fabrics/films, and 3D gels, while integrating functional characteristics such as stretchability, self-healing, waterproofness, breathability, biocompatibility, and high sensitivity [89]. These advances highlight the transition of triboelectric wearable sensors from simple self-powered sensing units toward intelligent and human-centered wearable electronics.

### Development of Triboelectric Sensor

Among the various self-powered sensors, triboelectric sensors have recently attracted widespread attention worldwide. The triboelectric sensor is fundamentally derived from TENG, which is an energy harvesting technology first reported by Wang’s group in 2012 [90]. TENG generates electricity by converting the mechanical energy into the electrical energy. It is a novel strategy to supply sustainable power for various terminal elements in IoT systems with independence from electrical grid. TENG can generate electricity from sequential contact and separation of two different material surfaces when they vertically face or laterally slide each other. Based on its unique advantages including the simple working mechanism and wide range of available materials, TENG can achieve various functionalities and wide application fields [48, 78, 91–99]. And with its advantageous characteristics described above, TENG has also developed as a self-powered sensor. It has a simple working mechanism and can adopt various materials with proper features for futuristic sensory systems including flexibility, stretchability, and biocompatibility [100–104]. Furthermore, it can be established by diverse constructions including two-dimensional, three-dimensional, interlacing, and even liquid-containing structures [105–116]. Given these superior merits, the triboelectric sensor has progressed to a promising wearable sensor [94].

### Triboelectric Sensor Mechanism, Material Characteristics, and Process

**Fig. 6:**
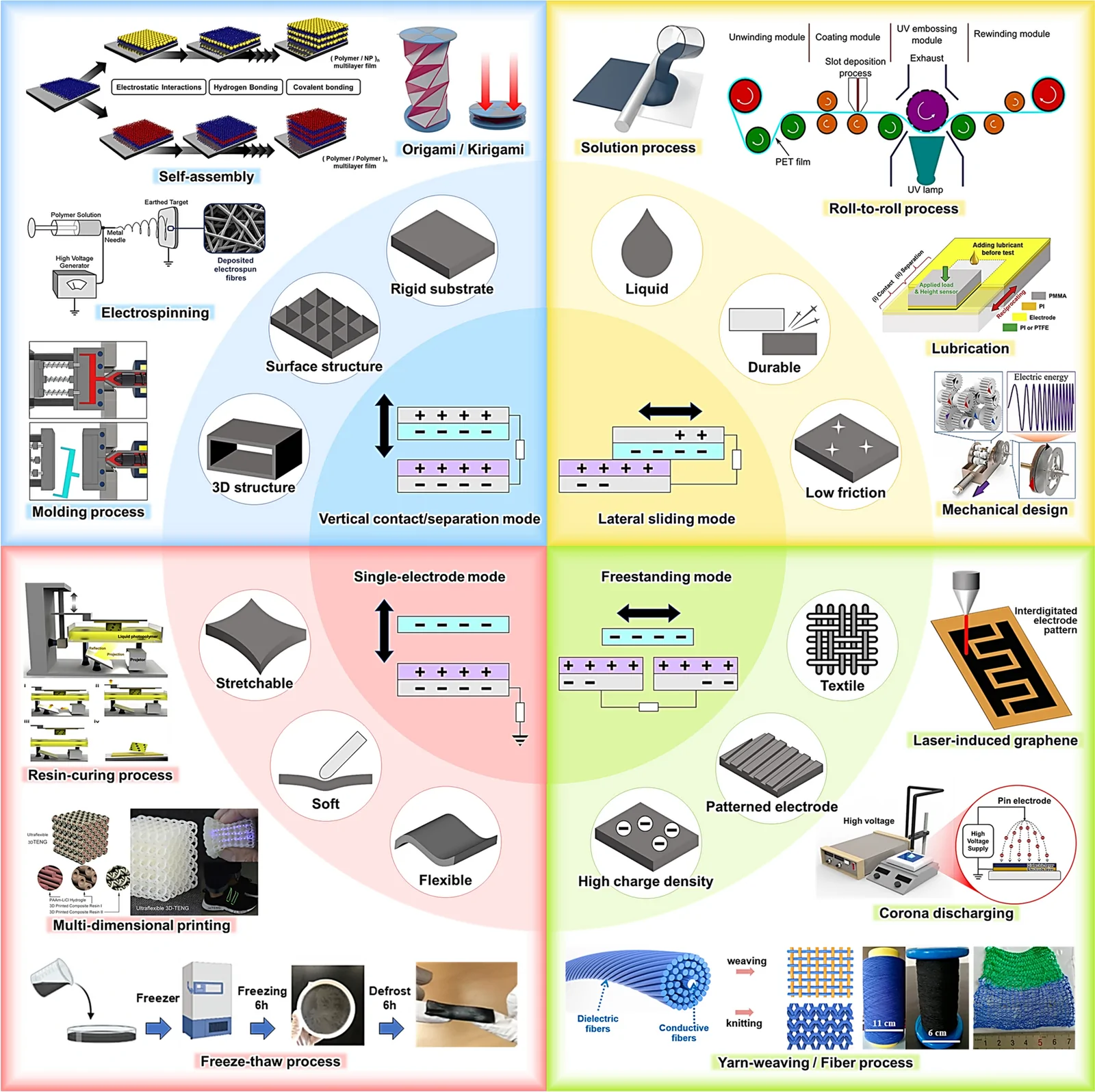
Operation modes, material characteristics, and fabrication processes of the triboelectric sensor. Reprinted with permission from Ref. [117]. © 2018 Elsevier Ltd. All rights reserved. Reprinted with permission from Ref. [118]. © 2021 Jihoon Chung et al. Reprinted with permission from Ref. [119]. Copyright © 2016, Dhakar, L., et al. Reprinted with permission from Ref. [120]. © 2020 Elsevier Ltd. All rights reserved. Reprinted with permission from Ref. [121]. © 2018 Lee, S., et al. Published by Elsevier Ltd. Reprinted with permission from Ref. [73]. © 2024 Elsevier Ltd. All rights are reserved, including those for text and data mining, AI training, and similar technologies. Reprinted with permission from Ref. [122]. © 2022 Elsevier Ltd. All rights reserved. Reprinted with permission from Ref. [123]. © Ra, Y., et al. 2025. Reprinted with permission from Ref. [124]. Copyright © 2023 Yoonsang Ra et al. Reprinted with permission from Ref. [125]. Copyright © 2014 Woodhead Publishing Limited. All rights reserved. Reprinted with permission from Ref. [126]. © 2020 Wu, J., et al. Published by Elsevier Ltd. Reprinted with permission from Ref. [127]. © 2019 Elsevier Ltd. All rights reserved. Reprinted with permission from Ref. [128]. Copyright © 2017, American Chemical Society

As is well known, the triboelectric sensor inherits the features of TENG such as the fundamental principle, material characteristics, working mechanism, operation modes, and fabrication process. Figure 6 presents the operation modes of the triboelectric sensors, related characteristics of used materials in each mode, and previously reported fabrication processes for them [73, 117–128]. The triboelectric sensor can be operated with four types of operation modes, vertical contact/separation, lateral sliding, single-electrode, and freestanding modes [37]. The vertical contact/separation and single-electrode modes can be applied to the case when two surfaces face each other. The lateral sliding and freestanding modes can be used when the two surfaces are rubbed against each other [129]. The vertical contact/separation and lateral sliding modes adopt two electrodes connected to each other [130]. The single-electrode and freestanding modes employ the electrode in only one of the contact layers. In this regard, the rigid substrate, surface structure, and three-dimensional structure can be available to construct the vertical contact/separation mode. Therefore, the self-assembly [131], origami/kirigami [132], electrospinning [133], and molding-based processes [124] are often used to fabricate the vertical contact/separation mode triboelectric sensor. In the lateral sliding mode, a low frictional and durable contact surface is considered a major factor for its higher performance. Given that, liquid material-based solution process [134], film-based process like roll-to-roll [135], and lubrication process [136] can be employed. Moreover, mechanical design is known as a potential strategy for efficient rotational operation and durability of components because the lateral sliding mode can effectively achieve high performance by rotational movement of components [137]. The single-electrode mode triboelectric sensor can include the material characteristics of stretchability, softness, and flexibility by employing resin curing [138], multidimensional printing [139], and freeze–thaw processes [69]. The single-electrode mode is one of the most primarily used in the triboelectric sensor. Because it can be effectively established by the most simple construction with the reduction of electrodes and wires, the single-electrode mode triboelectric sensor is widely applied to wearable electronics and IoT systems [140]. The freestanding mode does not include direct contact between the two material surfaces. It has airy space between the two surfaces, and therefore, the high-level charge density of the surface can be effective. Given that, many researchers have introduced the corona discharging method to accumulate net charges on the target surface [141]. Moreover, the textile structure and patterned electrode can be useful in this mode because it can work by sliding-like method. In this respect, the laser-induced graphene [142] and yarn-weaving/fiber processes [143] can be adopted. The material characteristics and fabrication processes are not only limited by each operation mode and various design strategies of the triboelectric sensor are possible for desired operation in optimal application. The expandability is one of the strongest points of the triboelectric sensor as a promising sensory technology for wearable electronics and future HMIs.

## AI-Processing Bridges from Triboelectric Wearable Sensing to Adaptive HMI: Artificial Synapses and Neuromorphic Computing

Triboelectric wearable sensors are highly attractive as front-end interfaces for human-centric smart electronics because they can directly convert human motion and physiological activities into self-powered electrical signals. However, the practical value of these devices does not lie in signal generation alone. Their outputs are often transient, nonlinear, user-dependent, and multimodal, which makes direct interpretation challenging when high-fidelity recognition, personalization, and real-time feedback are required in advanced human–machine interfaces. Therefore, a key question for this review is not only how triboelectric wearable sensors detect stimuli, but also how their self-generated signals can be translated into perception, memory, decision-making, and adaptive control. In this regard, artificial synapses and neuromorphic computing are highly relevant because they provide a biologically inspired and energy-efficient route for processing triboelectric sensory information beyond conventional readout electronics. Triboelectric-based artificial synapses can convert mechanically triggered signals into synaptic responses that emulate learning, memory, and plasticity, thereby enabling hardware-level preprocessing of sensory inputs. Neuromorphic computing further extends this concept by allowing event-driven, low-latency, and low-power interpretation of complex triboelectric signals in forms that are more compatible with real-world HMI operation than traditional von Neumann processing. For wearable systems, this is particularly important because it reduces data burden, improves real-time adaptability, and supports continuous interaction under power limited conditions. Accordingly, artificial synapses and neuromorphic computing are discussed here as enabling architectures that transform triboelectric wearable sensors from passive signal generators into active intelligent interfaces for next-generation HMI systems.

### Triboelectric Artificial Synapses and Neuromorphic Computing for Future Adaptive Intelligent Systems

Triboelectric artificial synapses have emerged as a class of sensing devices that integrate the principles of triboelectricity and artificial neural synaptic functions. Inspired by the sophisticated sensory processing and adaptability of biological skin and nervous systems, these sensors transduce mechanical stimuli into electrical signals with further memory and learning, inherently facilitating energy-efficient operations suitable for intelligent perception and AI-driven systems [144]. By closely mimicking the synaptic plasticity mechanisms found in human tactile neurons including excitatory postsynaptic currents (EPSCs), paired-pulse facilitation (PPF), short-term potentiation (STP), and long-term potentiation (LTP), these sensors achieve adaptive and comprehensive perception. Furthermore, with the integration of machine learning algorithms, the functionality and efficiency of the sensing system can be further enhanced. This fusion simplifies the traditionally complex system architecture, eliminates the need for extensive sensor arrays, and reduces energy consumption by utilizing self-generated electrical signals. Such capabilities represent a substantial advancement toward humanlike awareness of sensors and offer tremendous potential in the fields of robotics, wearable healthcare systems, human–machine interaction, and intelligent sensing platforms, effectively bridging the gap between artificial sensory systems and AI applications.

**Fig. 7:**
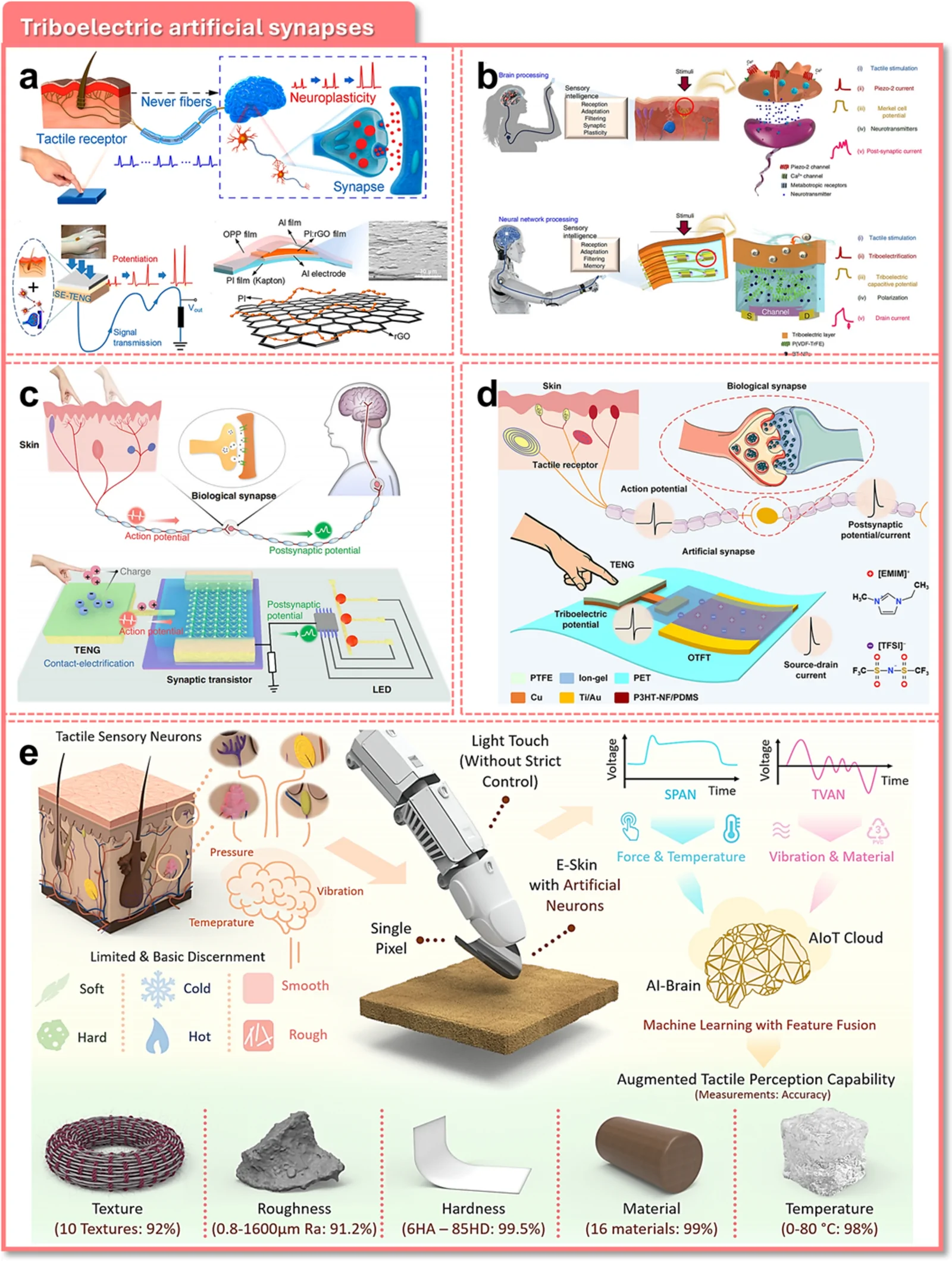
Triboelectric artificial synapses. **a** Artificial self-powered tactile sensor with learning and memory. Reprinted with permission from Ref. [145]. Copyright © 2020, American Chemical Society. **b** A flexible artificial intrinsic-synaptic tactile sensor. Reprinted with permission from Ref. [146]. Copyright © 2020, Yu Rim Lee et al. **c** Contact electrification-activated artificial afferents. Reprinted with permission from Ref. [147]. Copyright © 2021, Jinran Yu et al. **d** Biological afferent nervous system-based TAS device. Reprinted with permission from Ref. [148]. Copyright © 2022, Jianhua Zeng et al. **e** Zero-biased bionic fingertip e-skin with multimodal tactile perception. Reprinted with permission from Ref. [149]. © 2024 Wiley‐VCH GmbH

Wu et al. introduce a self-powered intelligent tactile sensor leveraging a single-electrode-based triboelectric nanogenerator (SE-TENG) that mimics synaptic functionalities such as learning and memory, akin to biological mechanoreceptors and neural systems as shown in Fig. 7a [145]. The sensor consists primarily of a polyimide: reduced graphene oxide (PI: rGO) hybrid nanocomposite layer, where rGO sheets are integrated into PI serving as electron body traps to facilitate neuromorphic behavior. Upon mechanical stimulation, the device actively generates voltage signals without external power, with a waveform resembling biological action potentials. The tactile sensor demonstrates synaptic potentiation behavior as successive mechanical stimulations progressively increase the generated output voltage from an initial 0.3 V to approximately 5 V at saturation after about 42 presses under constant pressure of 400 Pa and a short pressure-holding time of 0.05 s. The SE-TENG exhibits synaptic learning by integrating individual voltage increments (ΔEPSP) from repeated stimulations, demonstrating STM and LTM characteristics through controlled material and structural designs. The negative friction layers involve PI and rGO, wherein rGO sheets act as electron traps, enhancing electron transfer efficiency during repeated mechanical stimuli. Two types of devices were studied: INS-I with a single-layer PI:rGO film and INS-II with a stacked PI:rGO/PI layer, which demonstrates adjustable memory retention times corresponding to short-term and long-term memory, respectively. The INS-I shows relaxation times of approximately 70 min and 519 min for short-term and prolonged memory phases, while INS-II demonstrates a significantly extended LTM with a relaxation time of about 3082 min. The device's memory performance closely resembles human cognitive processes such as rapid forgetting followed by gradual retention loss. Furthermore, the sensor's capability to store and spontaneously release memory allows it to capture current as well as historical stimulation information. Demonstrations of practical utility included integration onto human fingers, successfully distinguishing various finger activities, and accurately reflecting past tactile interactions. This integration of triboelectric sensing and neuromorphic functionalities into a single device advances tactile sensor technology, enabling more sophisticated and energy-efficient sensing suitable for artificial intelligence and HMI applications. Lee et al. present a flexible artificial intrinsic-synaptic tactile sensory organ (AiS-TSO) based on triboelectric–capacitive coupling, inspired by the Merkel cell–neurite complexes in human somatosensory systems as shown in Fig. 7b [146]. This design forms intrinsic-synaptic connections to afferent neurons, enabling complex tactile preprocessing functionalities at a sensory organ level. The AiS-TSO enables direct emulation of intelligent biological tactile sensing capabilities including reception, adaptation, filtering, and memory. The primary structure of the sensor comprises a flexible ferroelectric organic field-effect transistor utilizing a gate dielectric layer composed of a nanocomposite with barium titanate nanoparticles (BT NPs) embedded within poly(vinylidene fluoride–trifluoroethylene) P(VDF-TrFE). The advancement presented in this work is the intrinsic integration of synaptic functions directly within the tactile sensing unit, rather than employing discrete sensors and processing components. Specifically, the triboelectric–capacitive coupling effect, induced by mechanical finger touch, modulates ferroelectric dipole alignment within the gate dielectric layer, controlling channel conductance and generating synaptic signals termed PSC. This modulation closely emulates biological tactile sensory preprocessing mechanisms. Experimental results demonstrate that synaptic functions such as STP and LTP were achieved and tuned by varying stimulation parameters including applied force, duration, and frequency of tactile stimuli, as well as adjusting the nanocomposite composition of the ferroelectric dielectric layer. For instance, the PSC increases significantly with applied pressures (0.3 to 3 kPa), stimulus durations (1 to 10 s), and frequencies (0.5 to 5 Hz). Synaptic weight (SW), defined as the ratio of change in PSC, is effectively tuned by controlling the concentration of BT NPs in the nanocomposite, enabling intelligent signal filtering and memory functionalities. Devices containing 40 wt% BT NPs achieve higher SW (approximately 3.8) at a longer touch duration (10 s) compared to lower concentrations or pure polymer layers, thus clearly distinguishing meaningful signals from noise. Additionally, the sensor array can memorize the sequence and number of touches without the requirement of external signal processing, thus mimicking biological sensory memory transitions from sensory to short-term and eventually long-term memory states. This integration offers significant advantages for AI applications by enhancing energy efficiency and reducing the complexity of processing systems, facilitating their potential usage in neurorobotics, intelligent electronic skins, and autonomous AI-based decision-making systems. Yu et al. describe an artificial afferent neuron activated by triboelectric contact electrification at ultralow energy consumption, specifically femtojoule level (11.9 fJ per spike), designed to mimic biological sensory neurons and synapses for advanced neuromorphic sensing networks and AI-related applications as shown in Fig. 7c [147]. The core device structure comprises an ion-gel-gated molybdenum disulfide (MoS_2_) transistor coupled directly to a TENG, leveraging the intrinsic contact electrification phenomenon to self-activate synaptic functionalities. The transistor utilizes atomically thin MoS_2_ as the semiconductor channel and employs ion gel dielectric as the gating layer, enabling efficient triboelectric potential coupling and ultralow-power operation. The principle of operation relies on CE-induced triboelectric potentials acting as presynaptic input spikes, modulating channel conductance in the MoS_2_-based transistor through electric double-layer (EDL) formation within the ion gel electrolyte. Such modulation mimics synaptic plasticity phenomena observed in biological afferents, including EPSCs, STP, paired-pulse facilitation (PPF), and dynamic logic functions, which collectively emulate the adaptive recognition of spatiotemporal tactile information such as displacement, pressure, and touch patterns. Specifically, the device demonstrates synaptic responses with measurable EPSCs reaching approximately 0.34 nA at a small displacement of 20 µm, with a decay time of around 54 ms corresponding closely to sensory memory processes. The synaptic characteristics exhibit an EPSC enhancement ratio (PPF index) controlled by the inter-spike interval (ranging from 1 to 4 s). Under repetitive stimuli (60 pulses), EPSC significantly increases from an initial value of 0.03 nA to approximately 9.7 nA, demonstrating short-term potentiation characteristics that closely mimic biological synaptic plasticity. Moreover, dynamic logic capabilities were experimentally validated using dual-gate configurations, enabling recognition of complex spatiotemporal stimulation patterns. The artificial neuron efficiently identifies multiple external stimuli based on variations in amplitude and frequency, confirmed through Fourier transform analysis of the recorded EPSC signals, thus enabling advanced AI-like functionalities for tactile sensing networks. Compared to previous studies, this work advances artificial synapse technologies by eliminating the requirement for external voltage sources, dramatically reducing energy dissipation, and offering integration of synaptic behaviors directly triggered by ambient mechanical stimuli. This approach helps to simplify the sensor–device interface and provides a robust platform for building distributed, self-powered neuromorphic sensory networks beneficial for applications in intelligent human–machine interfaces, robotics, and adaptive AI systems. Zeng et al. report a flexible tribotronic artificial synapse (TAS) device inspired by biological neurosensory systems as shown in Fig. 7d [148]. The TAS structurally combines an EDL-gated organic thin-film transistor (OTFT) with a TENG, which generates triboelectric potentials upon external mechanical stimuli. Specifically, the OTFT consists of a semiconductor layer made from poly(3-hexylthiophene) nanofibrils (P3HT-NF) and poly(dimethylsiloxane) (PDMS) composite, and an ion gel dielectric composed of poly(vinylidene fluoride-co-hexafluoropropylene) (PVDF-HFP) mixed with ionic liquid EMIM-TFSI. The integration of these materials provides superior mechanical flexibility, withstanding repeated bending cycles (bending radius of 20 mm) without significant degradation in device performance even after 1000 cycles. The TAS utilizes triboelectric potentials generated via contact electrification between copper (Cu) and polytetrafluoroethylene (PTFE) layers, directly serving as the gate voltage of the OTFT to modulate the carrier transport through ion migration within the ion gel electrolyte. This mechanism enables the TAS to exhibit synaptic functionalities that closely mimic biological synapses. Under a single mechanical stimulus (duration 0.1 s, displacement 2 mm), the TAS achieves EPSC peaks around 1.6 μA, which increase progressively with longer stimuli durations and higher repetition frequency. For multiple consecutive mechanical stimuli, the device exhibits EPSC peaks as high as 99.9 μA after 100 stimulations, demonstrating memory reinforcement akin to biological learning processes. Furthermore, the TAS successfully simulates associative learning through Pavlovian conditioning experiments, where vibration and mechanical force represent "bell" and "food" stimuli, respectively. Initially, the vibration stimulus alone does not surpass the threshold EPSC (set at 10 μA) required for triggering a salivation response, whereas, after repeated combined stimuli, the vibration alone can evoke significant EPSC responses, illustrating successful associative learning and memory retention. This bioinspired capability of associative learning enhances the potential applicability of TAS in intelligent systems, adaptive robotics, and advanced AI platforms by inherently integrating perception, learning, and memory functionalities within a single flexible device. Lastly, Guo et al., present a zero-biased, flexible electronic skin (e-skin) designed for multimodal tactile perception, effectively enhancing tactile awareness by integrating advanced AI for improved object identification and sensory interpretation as shown in Fig. 7e [149]. This bionic fingertip-inspired e-skin consists of two sensing components: a triboelectric-based transient voltage artificial neuron (TVAN) and an ionic hydrogel-based sustained potential artificial neuron (SPAN). The TVAN is structured using an Ecoflex film optimized with a honeycomb surface pattern, facilitating comprehensive texture, vibration, and material identification in a single pixel. The ionic hydrogel-based SPAN comprises polyvinyl alcohol/polyacrylamide (PVA/PAAm) hydrogels with lithium chloride (LiCl) and glycerol (Gly), capable of simultaneously detecting static pressure (ranging from 1 to 25 N) and temperature (ranging from 15 to 55 °C) without bias voltage and cross-signal interference commonly observed in prior sensors. Further advancement lies in synergistically integrating these sensors into a single fingertip unit and applying machine learning methods for feature fusion, significantly surpassing traditional tactile sensing systems that normally require complex arrays or separate sensors for tactile sensation. Specifically, the TVAN employs a triboelectric-based honeycomb pattern optimized for isotropic and uniform tactile sensation, yielding a high recognition accuracy of 92% for texture differentiation and up to 99.1% for material classification, outperforming linear or fingerprint-patterned devices. Concurrently, the SPAN sensor independently monitors pressure and temperature through ionic resistance modulation, demonstrated by distinct signal baselines and peak variations for each parameter. Leveraging deep learning, the combined signals from TVAN and SPAN allow the e-skin to recognize subtle variations in texture roughness (0.8–1600 μm) with 91.2% accuracy and hardness (6HA to 85HD) with an accuracy of 99.5%. Furthermore, through the synergistic use of TVAN and SPAN signals integrated via feature fusion, the e-skin is able to differentiate 16 distinct objects under varying temperatures (0–80 °C) with an average accuracy of 98.45% and independently identifies their respective temperatures with 98.85% accuracy. Its advanced holistic sensory perception is showcased in biometric user recognition, achieving identification accuracy of 97.8% across multiple individuals by capturing multifaceted biometric information beyond traditional fingerprint sensing like motion behavior, skin characteristics, and temperature. The comprehensive multimodal sensing capability of the proposed single-pixel e-skin inspired by artificial neurons, coupled with cloud-based AI, positions it ahead of traditional e-skins that rely on complex sensor arrays or single-mode sensing strategies, thus paving the way for more practical and widespread applications in smart robotics, health care, secure identification, and augmented HMI [150].

### Triboelectric Neuromorphic Computing Systems for Future AI

Neuromorphic computing has attracted growing attention because conventional von Neumann architectures are often not well suited for processing large volumes of continuous, multimodal, and temporally complex sensory data in real time [151]. In wearable and interactive systems, such data streams are frequently sparse, event-driven, and highly user-dependent, making repeated data transfer between sensing, memory, and computation units inefficient in terms of latency and power consumption. Neuromorphic computing offers an alternative by emulating biological neural systems, where information is processed through distributed synaptic weighting, spike-based communication, and adaptive plasticity. In this way, neuromorphic systems can perform sensing, memory, and inference in a more tightly integrated and energy-efficient manner.

When it comes to triboelectric sensing, neuromorphic computing is especially meaningful because triboelectric sensors inherently generate self-powered, transient, and event-like electrical outputs in response to external mechanical stimuli. These signal characteristics are naturally compatible with neuromorphic architectures, which are designed to process asynchronous and spikelike inputs rather than continuous analog streams. As a result, triboelectric-based neuromorphic systems can reduce data redundancy, lower computational burden, and improve real-time responsiveness while maintaining the advantages of flexible and wearable sensor formats. Therefore, the development of triboelectric neuromorphic computing is not merely an extension of artificial synapse research, but a promising route toward integrated intelligent systems that combine sensing, preprocessing, memory, and adaptive response within a unified self-powered platform. Such capability is particularly attractive for future applications in human–machine interfaces, robotics, intelligent healthcare devices, and interactive smart electronics. Recent studies have further reinforced the relevance of deep learning in triboelectric neuromorphic and intelligent sensing systems. Zhang et al. recently reviewed deep learning-enabled real-time visual monitoring of triboelectric nanogenerators and clarified the distinct roles of CNN, RNN, and LSTM architectures in denoising, feature extraction, and real-time signal interpretation. In addition, Liu et al. demonstrated programmable triboelectric origami sensors for multidimensional pressure monitoring, in which deep learning-assisted analysis enabled a recognition accuracy of 97.8%, further highlighting the growing role of intelligent triboelectric architectures in advanced sensing and robotic interaction [152–154].

Although artificial synapses have been extensively studied as fundamental building blocks capable of mimicking essential neural plasticity behaviors, integrating these components into comprehensive neuromorphic systems for practical AI remains challenging. Triboelectric-based neuromorphic computing systems have emerged as a particularly promising pathway for future AI, leveraging their unique self-powered characteristics and adaptive responsiveness to external mechanical stimuli. These systems combine triboelectric sensors, which effectively convert mechanical energy into electrical signals, with artificial synapses to achieve real-time, multimodal signal processing analogous to the human sensory and neural integration processes. The intrinsic advantages of triboelectric neuromorphic systems, such as low energy consumption, flexibility, high sensitivity, and autonomous sensing, enable them to seamlessly emulate tactile perception and synaptic transmission. Furthermore, integrating such devices with neuromorphic computing platforms facilitates advanced cognitive functions, including pattern recognition, sensory data interpretation, and adaptive learning. This convergence opens pathways to next-generation AI applications, spanning interactive HMI, intelligent robotics, and immersive mixed reality experiences, thereby significantly enhancing the interaction between machines and their dynamic environments.

**Fig. 8:**
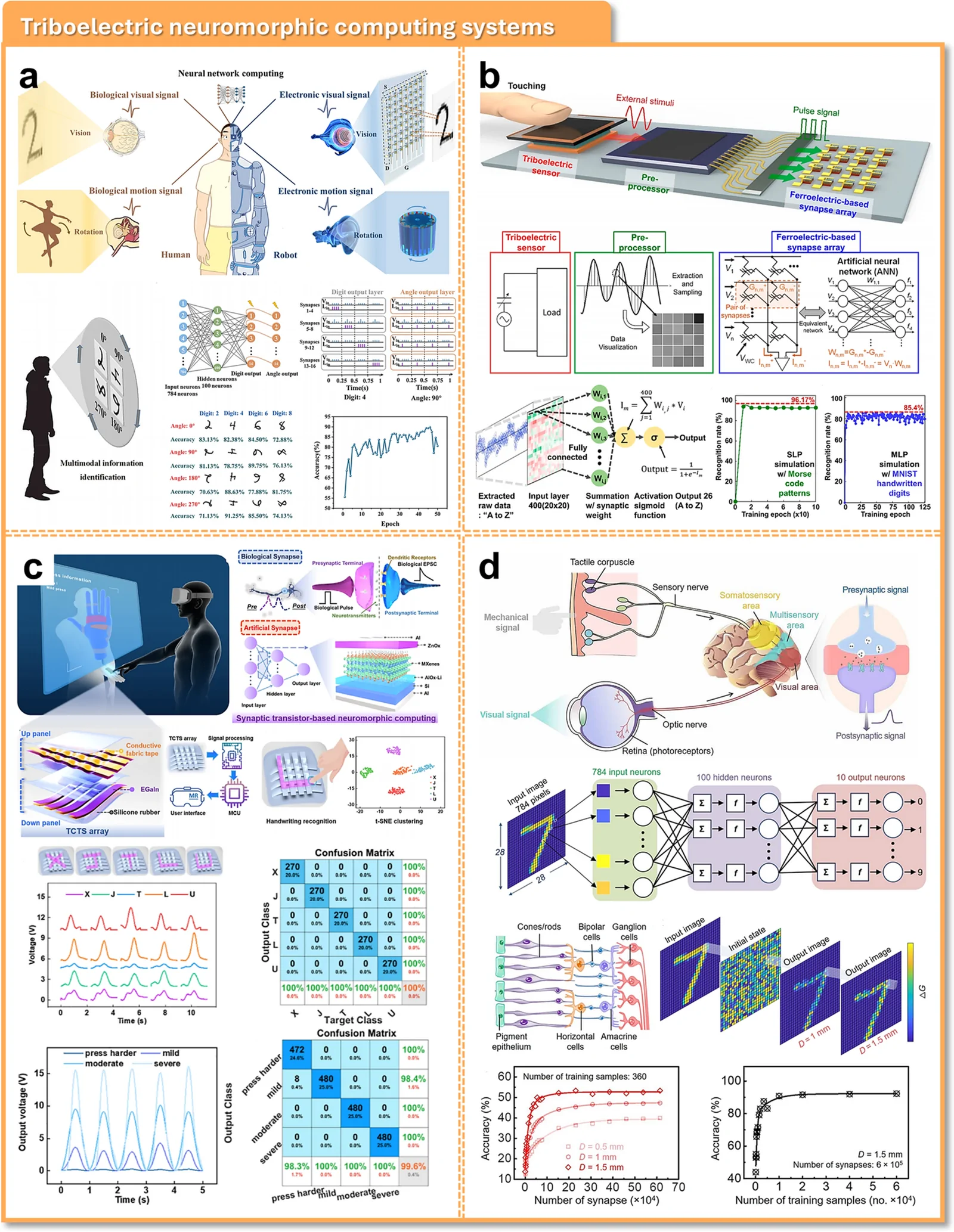
Triboelectric neuromorphic computing systems. **a** Bioinspired artificial motion sensory system for rapid self-protection. Reprinted with permission from Ref. [155]. Copyright © 2022, American Chemical Society. **b** Tactile neuromorphic system based on triboelectric polymer sensor. Reprinted with permission from Ref. [156]. Copyright © 2023, American Chemical Society. **c** Neuromorphic computing-assisted triboelectric–capacitive-coupled tactile sensor array. Reprinted with permission from Ref. [157]. © 2024 American Chemical Society. **d** Biological tactile/visual neurons and mechano-photonic artificial synapse. Reprinted with permission from Ref. [158]. Copyright © 2021, The American Association for the Advancement of Science

Chen et al. present a self-powered artificial motion sensory system (AMSS) inspired by the integration of visual and vestibular perception in biological organisms, specifically designed to enable advanced neuromorphic computing through multisensory integration as shown in Fig. 8a [155]. The proposed system combines a TENG, simulating vestibular input by detecting rotational movements, and an artificial retina constructed from a synaptic transistor array that transforms optical signals into EPSCs. The synaptic transistors are fabricated using a blended semiconductor channel comprising indacenodithiophene–benzothiadiazole polymer and CsPbBr_3_ quantum dots, exhibiting broad optical responsiveness and stable long-term synaptic plasticity behaviors, including PPF and long-term potentiation/depression (LTP/LTD). The advancement achieved in this work involves the successful realization of temporal congruency—an essential principle underpinning multisensory neural integration—where synchronized visual and vestibular signals result in substantially enhanced neural activity. To effectively mimic biological sensory processing and decision-making, the authors employed machine learning algorithms and neuromorphic computing techniques, particularly multilayer perceptrons (MLP) and spiking correlated neural networks (SCNN). Experimental validations conducted on the MNIST digit recognition task show that the combined visual–vestibular inputs improve recognition accuracy from 69.67% (vestibular only) to 85.83%, reflecting a clear computational advantage arising from multisensory integration. Moreover, leveraging the capability of AMSS to simultaneously perceive visual and rotational stimuli, the specifically designed SCNN was implemented, encoding information through integrated spatiotemporal neuron activation patterns. This network architecture facilitates multimodal information recognition, such as numerical digit classification coupled with precise angular orientation identification, with an accuracy as high as 89.82%. Compared with existing neural network frameworks employing single-mode sensing or purely spatial coding strategies, this approach demonstrates better performance due to its intrinsic ability to harness temporal relationships between different sensory inputs. Furthermore, practical tests validate that this neuromorphic system substantially accelerates decision-making processes critical for rapid self-protection scenarios, exemplified through robotic manipulation tasks simulating human protective reflexes. Reaction times decreased markedly—from approximately 6 s using only vestibular perception to 2 s upon integrating visual–vestibular modalities—highlighting the functional and computational superiority provided by the proposed multisensory neuromorphic approach. By effectively integrating sensory signal detection, synaptic plasticity simulation, and biologically inspired neural encoding, the AMSS contributes toward the development of neuromorphic computing systems capable of processing complex multimodal information. The improved computational efficiency and enhanced accuracy underscore its benefits in advancing AI applications, especially in robotics, prosthetic technologies, and intelligent soft electronics. Kim et al. describe a tactile neuromorphic system designed for real-time sensory data processing, specifically by integrating a PDMS-based triboelectric sensor with a ferroelectric polymer synapse composed of MoS_2_ and P(VDF-TrFE) as shown in Fig. 8b [156]. The goal of this system is to emulate human tactile sensory functions within a hardware-based artificial neural network (ANN), thereby enabling more effective real-time data processing. In the proposed tactile neuromorphic system, the triboelectric sensor, constructed from a Cu/PDMS/Cu structure, translates tactile stimuli into electrical signals that closely mimic human mechanoreceptors. Real-time measurements show that mechanical pressing resulted in positive voltage pulses ranging from 1.86 to 2.44 V with pulse widths of approximately 12 ms while releasing stimuli generated negative voltage pulses ranging from − 0.26 to − 0.46 V with pulse widths of about 50 ms. These tactile signals were processed and converted into visualized 20 × 20-pixel patterns for further analysis and recognition tasks. To replicate neural functionality, an artificial synapse was fabricated utilizing a ferroelectric field-effect transistor (FeFET) structure consisting of a MoS_2_ channel and a ferroelectric gate dielectric layer made from P(VDF-TrFE). The ferroelectric polymer provides nonvolatile polarization switching, enabling the device to exhibit both long-term potentiation and depression. Specifically, EPSCs and inhibitory postsynaptic currents (IPSCs) were modulated via external voltage pulses, demonstrating a maximum dynamic range of 78 and symmetricity of 4.7 under optimized conditions. These synaptic characteristics are critical for accurately training hardware ANN by effectively updating synaptic weights through electrical stimuli. For neuromorphic computing validation, the proposed tactile neuromorphic system utilized ANNs, specifically single-layer perceptron (SLP) and multilayer perceptron (MLP) architectures, to perform training and recognition tasks using Morse code alphabets and MNIST handwritten digit data. The SLP with an input layer size of 400 (representing 20 × 20-pixel tactile image patterns) and an output layer of 26 neurons corresponding to alphabet classification, achieved a recognition accuracy as high as 96.17%. Simulations also incorporated randomness to the Morse code dataset to prevent overfitting during ANN training. Furthermore, an MLP consisting of a 784-neuron input layer, a 100-neuron hidden layer, and a 10-neuron output layer was implemented and tested on the MNIST handwritten digits dataset, achieving a recognition accuracy of 85.4%. These performances illustrate significant computational effectiveness and validate the applicability of the MoS_2_/P(VDF-TrFE)-based FeFET synaptic devices in complex pattern recognition tasks typically encountered in neuromorphic computing applications. The practical neuromorphic tactile system demonstrated by this work through combining real-time sensing with neuromorphic synaptic functionalities, lays the groundwork for future hardware implementations of sensory–neuromorphic systems capable of handling complex real-world data efficiently in real-time AI applications. Xie et al. introduce a neuromorphic computing-assisted triboelectric–capacitive-coupled tactile sensor (TCTS) array tailored specifically for wireless mixed reality (MR) interaction, integrating dynamic and static tactile sensing capabilities as shown in Fig. 8c [157]. The system employs a dual-mode sensing approach, combining a capacitive sensor array for static tactile pressure mapping and a triboelectric sensor array for dynamic pressure variation detection. The TCTS array comprises two flexible electrode layers cross-stacked to form a 4 × 4 sensor matrix, utilizing liquid metal (EGaIn) as conductive electrodes encapsulated by silicone rubber, enabling flexibility and adaptability. Each sensor pixel measures approximately 7 mm, achieving high spatial resolution and providing sensitive detection down to 0.8 Pa with a rapid response time of about 6 ms. The operational principle of the TCTS involves two distinct sensing modes. In the static capacitive mode, applied pressure modulates capacitance values within each sensor unit, which change from approximately 5 to 19 pF under a load range of 0–80 kPa. The sensor exhibits a particularly high sensitivity of 17% kPa⁻^1^ within the low-pressure regime (below 1 kPa). In the dynamic triboelectric sensing mode, tactile pressure variations are detected through triboelectrification-induced voltage generation, achieving an impressive sensitivity of 7.88 kPa⁻^1^ within the small pressure range (0–8.78 kPa). Additionally, the sensor demonstrates minimal cross talk among adjacent pixels and robust repeatability under various conditions, highlighting its reliability for practical applications. This research implements neuromorphic computing based on an MXene-based synaptic transistor. These synaptic devices are structured as Al/ZnO_x_/MXenes/AlO_x_-Li/Si/Al layers, effectively emulating biological synaptic behaviors. SW within ANNs are represented by modulating conductance states of these synaptic transistors, enabling hardware-based neuromorphic computation. For validation, the authors utilized SLP neural network architecture and successfully classified handwritten numbers and letters captured dynamically by tactile inputs. Handwritten numerical data (digits 0–9) collected by the TCTS array were recognized with 100% accuracy within 90 training epochs, clearly indicating the computational efficiency and high performance of the proposed neuromorphic computing framework. The dimensionality of complex tactile data sets was effectively reduced using t-distributed Stochastic Neighbor Embedding (t-SNE), confirming clear differentiation between signal clusters corresponding to different handwriting inputs or pressure levels. Furthermore, the authors validated practical applications by developing an MR-based HMI. Real-time tactile data captured by the TCTS was processed via analog-to-digital conversion and wirelessly transmitted through Bluetooth to an MR visualization interface developed in Unity. This facilitated real-time visualization of tactile interactions, such as the strength and distribution of pressure, providing intuitive, immersive user experiences applicable to telemedicine, physiotherapy, and advanced HMI. The presented system therefore represents a progression in tactile neuromorphic computing, integrating multimodal sensory detection with neuromorphic processing, ultimately contributing toward practical applications in intelligent robotics, AI-enabled interfaces, and immersive MR platforms. Yu et al. present a bioinspired mechano-photonic artificial synapse based on a graphene/MoS₂ heterostructure aimed at advancing neuromorphic computing through synergistic mechanical and optical plasticity as shown in Fig. 8d [158]. The system architecture integrates an optoelectronic transistor and a TENG, effectively emulating biological synaptic functions. Specifically, the artificial synapse comprises a graphene/MoS₂-based optoelectronic transistor integrated with a Cu/PTFE/Cu contact–separation TENG. The TENG generates triboelectric potentials in the range of approximately + 62 to − 52 V under mechanical displacements between − 1.75 and 1.75 mm, serving as a gate modulation source to control charge transfer between graphene and MoS₂ layers. The graphene/MoS₂ heterostructure functions by transferring photogenerated charge carriers in MoS₂ to graphene via electrostatic band bending, enabling persistent photoconductivity crucial for neuromorphic behavior. The optoelectronic characteristics demonstrate tunable photosensitivity between 2.4 × 10^4^ and 9.3 × 10^5^ A W^−1^, directly modulated by mechanical displacement from 0.5 to 1.5 mm under a fixed illumination power of 11.5 mW cm^−2^. Such displacement-controlled optoelectronic tuning represents significant progress compared to prior unimodal neuromorphic devices, enabling dual-modal plasticization via mechanical and optical stimuli simultaneously. The mechano-photonic synaptic behavior was investigated by applying mechanical displacement alongside optical stimuli (green LED, 525 nm wavelength). Postsynaptic photocurrents exhibited a distinct modulation from 9 µA at 0.75 mm displacement to 24 µA at 1.5 mm displacement under consistent optical excitation (3.5 mW cm^−2^, 50 ms pulses). The synaptic plasticity exhibited long-term depression-like behaviors with persistent photoconductivity and tunable retention exceeding one hour. The synaptic plasticity is attributed to the triboelectric potential-induced modulation of the graphene Fermi level and MoS₂ energy band alignments, controlling electron injection and recombination processes. Specifically, displacement-induced triboelectric potentials shift carrier populations within the heterostructure, creating persistent charge states essential for synaptic emulation. Furthermore, ANN simulations incorporating these synaptic characteristics demonstrated substantial enhancements in image recognition accuracy. A three-layer ANN (784 input neurons corresponding to 28 × 28 pixels, 100 hidden neurons, and 10 output neurons) trained on the MNIST dataset achieved recognition accuracy improvements from 37 to 54% as displacement varied from 0.5 to 1.5 mm with only 360 training samples. With increased training samples (up to 60,000), accuracy further improved significantly to 92%, underscoring the efficacy of mechanical plasticization in neuromorphic learning applications. This work represents considerable progress in multimodal neuromorphic computing, providing foundational advancements toward highly interactive, flexible neural networks capable of sophisticated sensory integration.

## Triboelectric Wearable Sensors with AI in Personalized Interactive Technology

As technology moves toward hyper-personalization, the demand for seamless, intuitive, and self-sufficient interactive systems continues to grow. In this regard, triboelectric wearable sensors are poised to become a cornerstone of personalized interactive technology, enabling direct and real-time interaction between humans and devices without external power constraints. These sensors offer an ideal solution for applications such as healthcare monitoring [159], gesture recognition [160], and intelligent device control [161], where continuous and adaptive sensing is critical. A key advantage of triboelectric sensors is their ability to self-powered sensing through natural human motion, thanks to the triboelectric effect Triboelectric wearable sensors are likely to alleviate part of the need for batteries, ensuring sustainability, long-term operation, and uninterrupted real-time sensing. This makes such interactive applications more personalized and adaptive to individual users. Furthermore, integrating AI with triboelectric wearable sensors significantly expands their system-level capabilities. Rather than changing the intrinsic sensing mechanism itself, AI refines the downstream processing of triboelectric signals through denoising, feature extraction, temporal pattern recognition, multimodal fusion, and adaptive calibration, thereby improving classification performance, user-specific adaptability, and personalized feedback. This combination of self-powered sensing and AI-assisted intelligence expands the practical scope of applications including health care, smart gloves, virtual interaction, intelligent HMI, and robotics, because the sensor-generated signals can be more reliably converted into interpretable commands, recognized patterns, and context-aware responses.

### Health Care

**Fig. 9:**
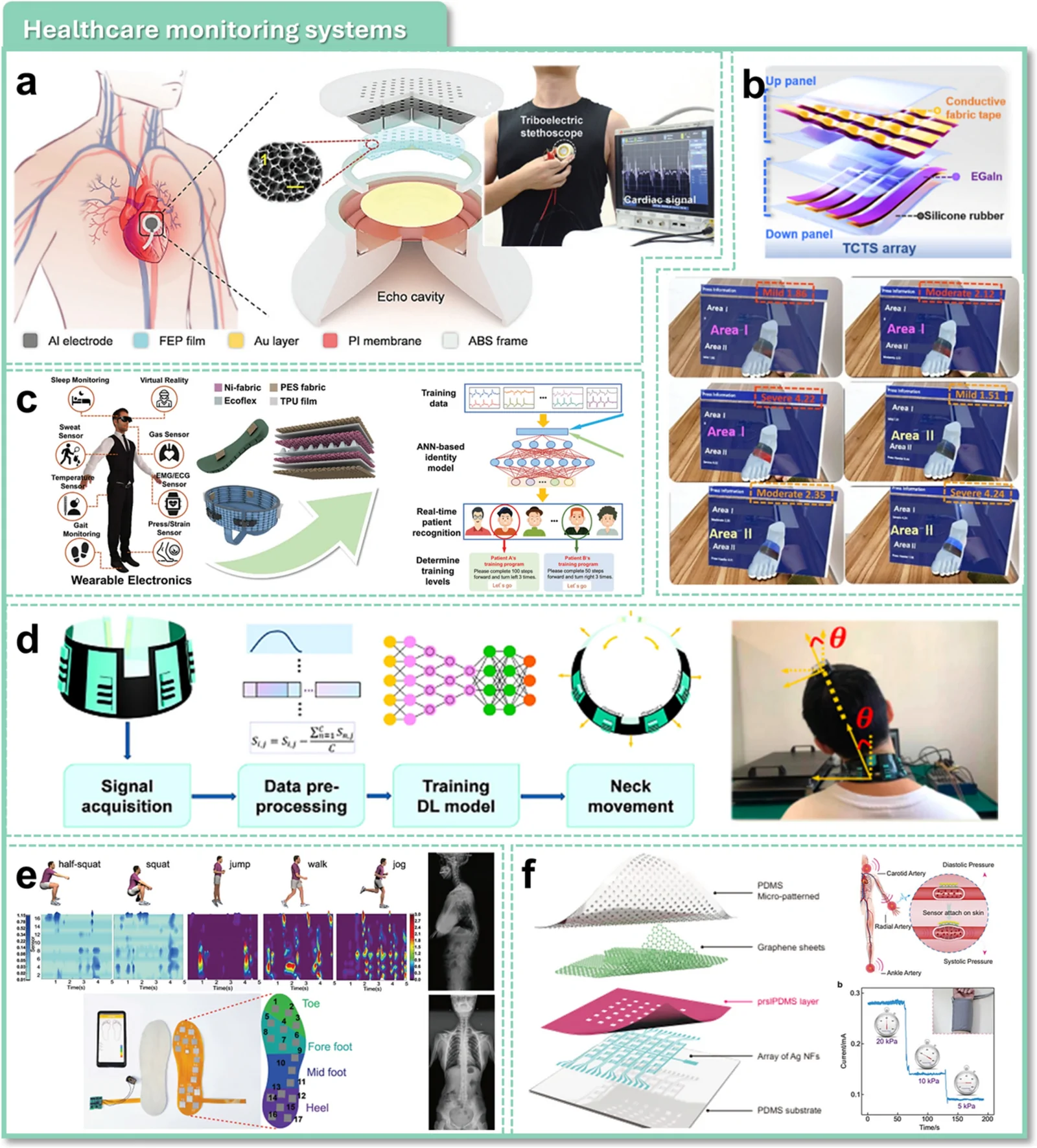
Applications of health care. **a** Triboelectric stethoscope for ultrasensitive cardiac sounds sensing. Reprinted with permission from Ref. [159]. © 2024 Wiley‐VCH GmbH. **b** Self-powered acupressure sensing. Reprinted with permission from Ref. [157]. © 2024 American Chemical Society. **c** Triboelectric wearable sensor enabled gait analysis and waist motion. Reprinted with permission from Ref. [163]. © 2021 Zhang, Q., et al. Advanced Science published by Wiley‐VCH GmbH. **d** Triboelectric sensor enabled neck motion detection. Reprinted with permission from Ref. [165]. Copyright © 2022, American Chemical Society. **e** Active-matrix sensing array for lumbar degenerative disease diagnosis. Reprinted with permission from Ref. [167]. © 2022 Wiley‐VCH GmbH. **f** Real-time detection of human pulse. Reprinted with permission from Ref. [168]. Copyright © 2023, Yufei Zhang et al.

In modern health care, continuous physiological monitoring is essential for disease prevention, early diagnosis, and patient management [162]. Triboelectric wearable sensors offer a game-changing solution by harvesting biomechanical energy from body movements to generate electrical signal in real-time health monitoring [163]. These sensors can track vital signs such as heart rate, respiratory patterns, and rehabilitation therapy [159]. Moreover, the AI-assisted triboelectric wearable sensor enables these devices to predict health risks with high accuracy. AI technologies can analyze long-term trends, allowing for personalized treatment recommendations and early detection of conditions. In this regard, Hui et al. reported a systematical investigation on the electromechanical output character of a contact–separation mode (C–S) TENG and propose its superior advantage with low-intensity and low-frequency cardiac sounds as presented in Fig. 9a [159]. The C–S TENG features more flexible design capabilities to further increase the sensitivity of heart sound detection by optimally incorporating low-frequency mechanical information without compromising electromechanical coefficients, demonstrating high sensitivity (1215 mV Pa^−1^) in the 50─80 dB sound pressure range. A power-law shaped auscultatory cavity is designed to optimize acoustic impedance matching, improving signal-to-noise ratio (SNR) by 2.3 times of 56 dB. Leveraging machine learning algorithms, the accuracy can be increased to 97% in diagnosing five cardiac states, contributing to the evolution of sophisticated medical equipment. Another is research into healthcare monitoring, not through the sensing of highly sensitive mechanical energy like heart rate but rather through the sensing of foot pressure, which is the weight-bearing capacity of the body. Xie et al. introduce a dual-mode flexible triboelectric–capacitive-coupled tactile sensor (TCTS) array by coated stripe electrodes and silicone rubber lamination, as shown in Fig. 9b [157]. The TCTS consists of 16 sensing units (4 × 4 array) with a total of 16 pixels for tactile pressure mapping and recognition. In triboelectric sensing mode, it can achieve 7.88 kPa^−1^ sensitivity (0─8.78 kPa range), with a 0.8 Pa detection limit and 6 ms response time. In addition, by integrating with the synaptic transistor-based neuromorphic computing for artificial neural network (ANN), the TCTS attains 100% accuracy in recognizing handwriting. Consequently, the TCTS array functions as a multichannel tactile sensor for acupressure intensity visualization, facilitating cross-spatial information communication in real-time mixed reality for individual therapy. While AI does not directly alter the intrinsic pressure sensitivity of the device, it improves the interpretation of acupressure data by enhancing classification accuracy and patient-specific pattern recognition, the next study uses AI to distinguish patients based on a personalized database [164]. As shown in Fig. 9c, Zhang et al. proposed a textile-based triboelectric sensory system for gait analysis and waist motion capture to improve the performance of the robot-aided lower limb and waist rehabilitation, which are representative therapies for patients [163]. They developed a universal type of textile-based triboelectric wearable sensor, which is structured by the pyramid-patterned layers by encapsulating with TPU-coated fabric via heat sealing for its robustness. To distinguish patients individually, the proposed sensors can be used on the waist, wrist, and insole. Each sensor can collect the database by recording different gait analyses, walking speeds, and conditions of each patient. In particular, the diversified information from insole TENGs (I-TENGs), the machine learning technique is applied to recognize the features of users and to establish rehabilitation plan selection with protecting personal privacy. Similarly, An et al. conduct a deep learning (DL)-enabled self-powered neck motion triboelectric sensor (SNM-TS) with various advantageous aspects of skin-friendly, flexible, stretchable, low-cost, and easy-to-fabricate neck motion, as presented in Fig. 9d [165]. Importantly a carbon-doped silicon rubber layer attached to the neck collar enhances the SNR. Therefore, the SNM-TS recognizes the motion state of the neck with the four channels stably by analyzing the generated voltage signals. Therefore, with an accuracy rate of 92.63% through AI technology, SNM-TS can be utilized to detect and correct cervical spine posture, rehabilitation training for neck diseases, and improving the lives of people with upper limb disabilities. Considering the above research, continuously analyzing a posture of person with the triboelectric wearable sensor can be of great help in preventing variety of diseases and illnesses [166]. Liu et al. investigated the method to prevent lumbar degenerative disease (LDD), in which the lumbar canal narrows and compresses the dural sac, cauda equian, or nerve root. They designed a wearable in-shoe monitoring system, which consists of a flexible insole with active-matrix sensing sports, and a data processing circuit board, as presented in Fig. 9e [167]. A support vector machine (SVM) is employed for AI recognition with the real-time plantar pressure mapping by active-matrix sensing array (AMSA). In particular, various human body motions including squat, half-squat, jump, walk and jog, which are helpful for diagnostic of LDD, leading to recognizing accuracy 100%. Thus, as AI-assisted triboelectric wearable sensor improves, individuals can be better equipped to prevent and respond to illnesses with the help of doctors based on the recorded database. Recently, there has been a lot of research into triboelectric wearable sensors capable of detecting pulse. Zhang et al. demonstrated the photoreticulated strain localization films (prslPDMS) [168]. The proposed ultrathin pressure sensor is configured by stacking the Ag NF, prslPDMS, and micropatterned graphene layer, thereby achieving high-pressure resolution to sense the 1g (~ 150 Pa) weight. Figure 9f reveals a human pulse detection ability by attaching to various arteries in human body, such as carotid, wrist, and ankle [168]. In this regard, the blood pressure and pulse are crucial for assessing cardiovascular health, and preventing serious conditions like heart disease and stroke, enabling early intervention and personalized treatment. Then, the typical pulse waveform was measured with three distinguishable peaks, such as systolic peak (*P*_*s*_), point of inflection (*P*_*i*_), and dicrotic wave (*P*_*D*_). In this section, AI-assisted triboelectric wearable sensors are showing promise in revolutionizing healthcare monitoring by enabling real-time, self-powered, continuous tracking of vital signs such as blood pressure, heart activity, and gait patterns. These studies could contribute to future medical advances by combining triboelectric energy harvesting with AI analytics to improve data accuracy, predictive diagnostics, and personalized health care, enabling early disease detection, remote monitoring, and intelligent health care without relying on external power sources.

### Gesture Recognition

**Fig. 10:**
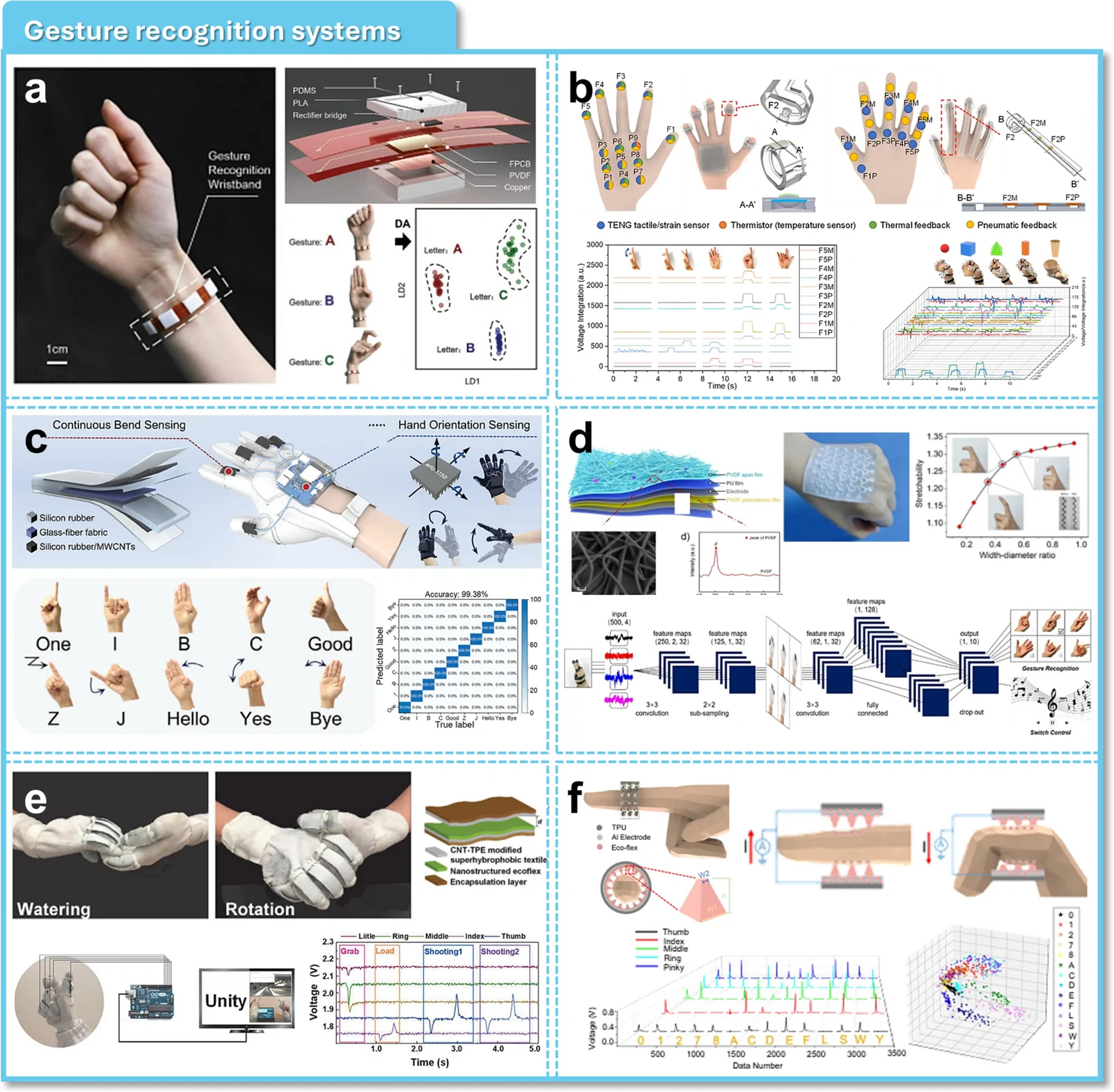
Applications of gesture recognition. **a** A self-powered gesture recognition wristband for full keyboard. Reprinted with permission from Ref. [169]. © 2022 Wiley‐VCH GmbH. **b** Smart modular glove for multimodal sensing and augmented haptic feedback. Reprinted with permission from Ref. [29]. Copyright © 2022, American Chemical Society. **c** Triboelectric-inertial sensing glove. Reprinted with permission from Ref. [170]. © 2024 Linhong Ji et al., Advanced Science published by Wiley‐VCH GmbH. **d** Self-powered tactile sensor using deep learning algorithms. Reprinted with permission from Ref. [160]. Copyright © 2022, American Chemical Society. **e** A Self-powered conductive superhydrophobic triboelectric textile sensor. Reprinted with permission from Ref. [171]. © 2020 Wen, F., et al. Published by WILEY‐VCH Verlag GmbH & Co. KGaA, Weinheim. **f** ATH-Ring-based intelligent system. Reprinted with permission from Ref. [172]. Copyright © 2022, Zhongda Sun et al.

Human–computer interaction has traditionally relied on physical interfaces such as keyboards, touchscreens, and cameras. However, these methods often have limitations in adaptability, precision, and privacy. Triboelectric wearable sensors provide an alternative by detecting minute biomechanical signals generated from hand and finger movements, enabling gesture-based interaction without direct physical contact [73]. Unlike camera-based systems, which are susceptible to lighting conditions and occlusions, triboelectric sensors function reliably in diverse environments. Moreover, AI-driven algorithms further enhance their performance by learning complex gesture patterns, adapting to different users, and reducing classification errors. In this regard, Tan et al. presented a gesture recognition wristband (GRW) based on the wristband-style sensor capable of realizing a full keyboard and multicommand input. The GRW has great advantages with convenient use, low cost, and high adaptability, considering the user experience, as exhibited in Fig. 10a [169]. Notably, since this gesture sensor is based on physiological anatomy as well as aided by active sensor and machine learning technology, the machine learning model achieves up to 92.6% recognition accuracy for 26 letters by learning user-specific signal patterns from the triboelectric wristband outputs. Therefore, with above features, GRW has the potential to be employed in various fields such as health care, biomedical engineering, military, aerospace, and as an assistive tool for people with disabilities. Recently, the advancement of multimodal sensors that can sense various conditions as well as measure simple hand gesture has been attracting attention. With respect to multimodal sensing, Zhu et al. developed a modular soft glove for multimodal sensing and feedback functions from the same unit, called Tactile^+^ (tactile plus) in Fig. 10b [29]. Tactile plus units consist of five-finger modules and a palm module, providing various types of conditions, such as triboelectric tactile and strain sensing, pneumatic actuation, triboelectric-based monitoring of pneumatic actuation, temperature sensing, and thermal feedback. Thus, the as-fabricated tactile plus exhibits its feasibility for the intelligent fusion of sensing and feedback capabilities, thereby enabling the mutual communication in virtual space among different users. With the help of these various capabilities, machine learning technologies, and IoT infrastructure, smart perception can be achieved by intelligently detecting motion and external stimuli and presenting a variety of stimuli with comprehensive information. In the process of advancing gesture sensing, Yang et al. presented the potential of both triboelectric wearable sensor and existing circuits [170]. In Fig. 10c, a triboelectric–inertial dual-mode sensing glove (TI-Glove) is proposed. The TI-Glove is configured by five triboelectric-based sensors for finger bending motion sensing, and even IMU for hand orientation detecting. This innovative integration of triboelectric sensor and IMU circuit enables real-time operation, precise sensing, and continuous motion recognition of hand motion with zero-power bending sensing with the advantages of multifunctionality and portability. Thus, combination of triboelectric-based sensor and traditional circuit for precise sensing provides new insights into the potential applications of future smart gloves in human–machine interaction and sign language communication. And then, there is even a study that encompasses many of the benefits mentioned above. Yang et al. presented a hybrid tactile sensor based on both triboelectric effect and piezoelectric effect with the advantageous characteristics for wearable device, such as multifunctional integrated, stretchable, and high-durability self-powered tactile sensor, as presented in Fig. 10d [160]. It is noteworthy that they fabricate the hybrid sensor by employing the electrospinning process with polyvinylidene fluoride (PVDF), a well-known material for its stability. Thus, The SNR is 22.5 dB, the response time is 5 ms, and the pressure resolution is 1%. In addition, the convolutional neural network (CNN) is used to recognize the gesture and interaction, providing meaningful results for its human–computer interaction, signal monitoring and intelligent sensing. Similarly, Wen et al. developed a research to increase the water stability as a wearable sensor by imposing a superhydrophobic coating on a textile-type sensor during exercising [171]. In Fig. 10e, a glove-based HMI with superhydrophobic-coated textile achieves to recognize complex gestures by training finger motion signals with machine learning with the accuracy of from 99.4% to 96.9% in sweat conditions. Therefore, this sensor shows great potential in sports applications, such as baseball pitching, shooting games, and floral arrangements. Additionally, to maximize user convenience, a ring-type wearable sensor is also being developed. Sun et al. showed augmented tactile perception and haptic feedback rings (ATH-Rings) with multimodal sensing and even feedback capabilities, in Fig. 10f [172]. They intended to fabricate minimalistic-designed ring with the merits of a high level of integration and excellent portability compared to other previously reported solutions. Additionally, we use sensor signals to operate integrated vibrators and heaters to achieve adjustable vibration and thermal tactile feedback to simulate the sensation of touching objects in virtual space. Therefore, the combination of advanced triboelectric wearable sensor, highly integrated circuit, and machine learning could accelerate the opening of interactive social, education, entertainment, and virtual social experience. This section explores the practical implementation of AI-enhanced triboelectric sensors in gesture-based interfaces and their role in assistive technologies, gaming, and augmented reality.

### Device Control

**Fig. 11:**
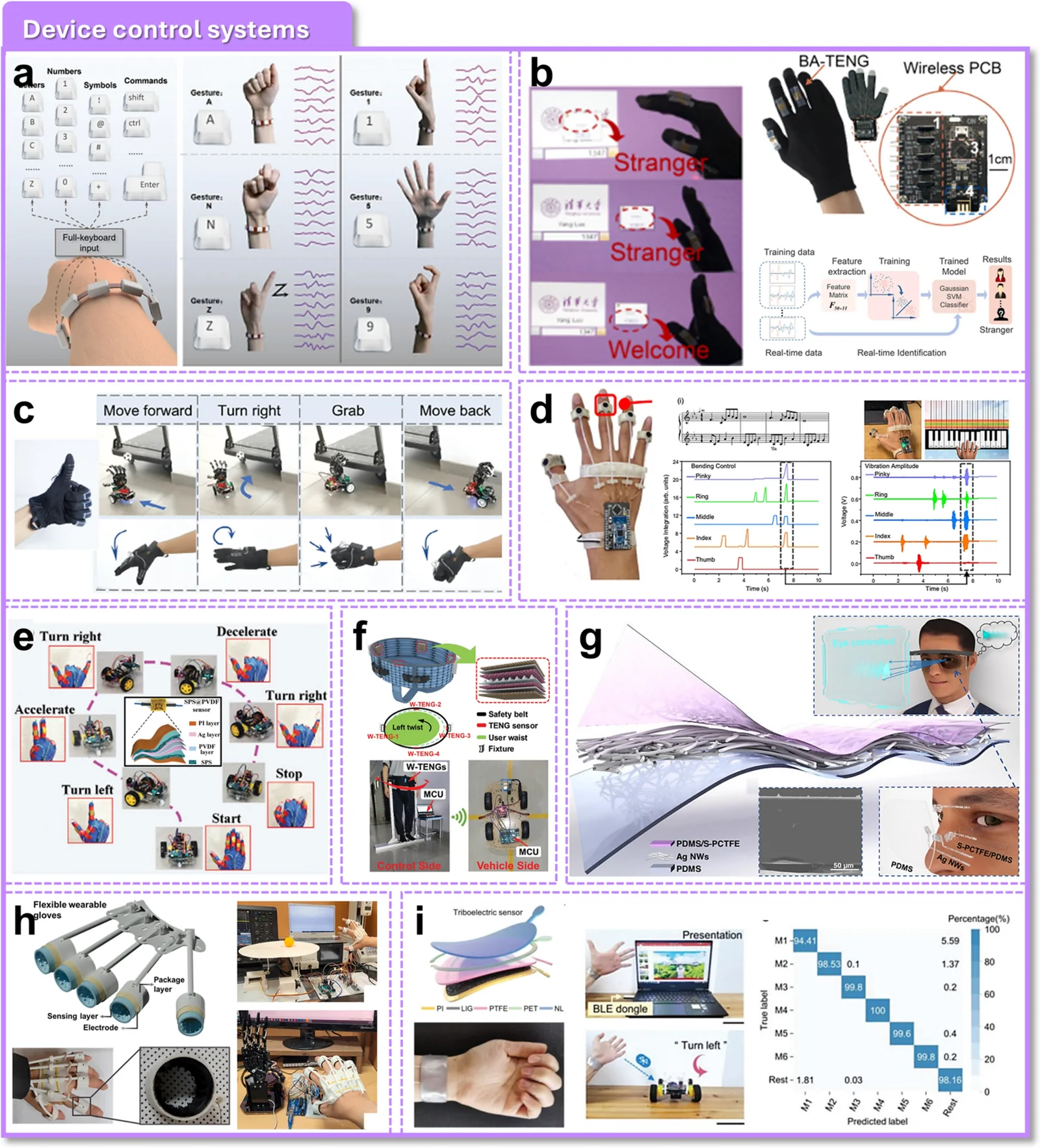
Applications of device control. **a** Smart gesture recognition sensor for universal sign language. Reprinted with permission from Ref. [169]. © 2022 Wiley‐VCH GmbH. **b** Smart glove based on triboelectric bending sensor. Reprinted with permission from Ref. [175]. © 2021 Elsevier Ltd. All rights reserved. **c** Playing virtual piano with multiple fingers. Reprinted with permission from Ref. [170]. © 2024 Linghong Ji et al., Advanced Science published by Wiley‐VCH GmbH. **d** A self-powered intelligent shooting system for improving police shooting technology. Reprinted with permission from Ref. [172]. Copyright © 2022, Zhongda Sun et al. **e** Toy cars control by recognizing hand gesture. Reprinted with permission from Ref. [176]. © 2024 Wiley‐VCH GmbH. **f** Smart belt based on TES to control the vehicle. Reprinted with permission from Ref. [163]. © 2021 Chengkuo Lee et al., Advanced Science published by Wiley‐VCH GmbH. **g** TENG-based electrostatic interface for sensing eye tracking. Reprinted with permission from Ref. [161]. Copyright © 2023, Yuxiang Shi et al. **h** 3D-printed flexible wearable glove assembled with STTS. Reprinted with permission from Ref. [177]. © 2022 Elsevier Ltd. All rights reserved. **i** Triboelectric smart wristband for HMI. Reprinted with permission from Ref. [178]. © 2023 Hao Wu et al., Advanced Science published by Wiley‐VCH GmbH

The increasing prevalence of smart devices necessitates intuitive and responsive control mechanisms [173]. Triboelectric wearable sensors offer a promising solution by converting human movement into electrical signals that can be used to control electronic systems. These sensors can detect subtle hand gestures, limb movements, or even facial expressions to trigger predefined actions in smart home devices, industrial automation, and wearable electronics. AI-driven signal processing further refines the accuracy of sensor responses by learning user-specific movement patterns, reducing false triggers, and enabling customized interactions. Tan et al. developed a sophisticated, highly integrated, and real-time gesture recognition wristband (GRW) that can be employed in the mechanical control, keyboard input, and other scenarios, as presented in Fig. 11a [169]. To demonstrate its ability to recognize the gesture, they performed the wristband as a keyboard input device to recognize the user’s real-time sign language gestures, convert them into sentences, and broadcast them out loud [174]. Moreover, the linear discriminant analysis (LDA) model is used to improve recognition accuracy up to 92.6% after 600 iterations of repeated training. On the other hand, Luo et al. reported a wearable glove-based system for real-time intuitive multidimensional HMI in Fig. 11b [175]. They developed a bending angle triboelectric nanogenerators (BA-TENG) to control many applications, such as light control, advanced robotic hand control, and a virtual keyboard. Furthermore, the BA-TENG demonstrates that it is possible to distinguish users by identifying their habits or patterns through machine learning algorithm with a high recognition rate of 93.1% against 7 different users. Similarly, Yang et al. suggested a novel solution with smart glove by leveraging the dexterity of human hands for applications including complex robotic control, surgical robotics, and smart factory [170]. This smart glove, which consists of triboelectric and inertial sensing unit, enables a well-balanced mix of versatility and system simplicity considering the user experience in Fig. 11c. In particular, the potential utility of the proposed sensor in remote synchronous control of robot for rigid material handling. With this device, for example, employees can remotely guide the robot to a designated location with only hand gestures, synchronize more sophisticated motions to pinpoint the target object, and move the object to a safe place again. In addition, triboelectric wearable sensors can also be used to play virtual instruments such as the piano. Playing the piano is very complex and requires high accuracy of gesture recognition, and it should not interfere with the user's ability to play the piano when worn. In this regard, Sun et al. developed a ring-type wearable sensor that can interact with piano playing to make it highly immersive thanks to the machine learning (ML) analytics [163, 176]. The ring on each finger can independently monitor the gesture in real time, and return the specific vibration feedback after receiving the collision signal in the virtual space, presenting the next-generation educational training applications in the future. In addition, similar sensors can also be employed for mobility control, such as operating a toy car. Specifically, as shown in Fig. 11e, f, the fabricated sensor can be worn on the hand or attached to a waist belt, allowing the user to wirelessly control the movement of the car according to their intentions. Each hand motion has already be input in a predetermined control command, such as “start,” “left,” “right,” “accelerating,” “decelerating,” and “stop.” To increase the accuracy, convolutional neural network (CNN) and gated recurrent unit (GRU) are used for deep learning. Interestingly, Zhang et al. reported an active eye-tracking (AEF) sensor for eye movements monitoring and eye-controlled HCI in real time [161]. In addition, in Fig. 11g, the triple-layer structure with silver nanowires electrode, ultrathin dielectric layer of polychlorotrifluoroethylene (PCTFE) grafted onto PDMS, enables an amplified charge storage effect, maintaining an ultrapersistent electrostatic charge density over thousands operation cycles. This high stability allows the AET system to accurately decode eyelid movements induced by oculogyria and the corresponding skin fluctuations. Ultimately, a real-time eye movement and gaze tracking designed for visual preference analysis and commercial applications were demonstrated. Therefore, they presented an eye-controlled input modality aimed at assisting individuals with disabilities in achieving seamless HCI. To improve wearability of sensor, Zhang et al. fabricated a self-powered toroidal triboelectric sensor (STTS) based on an MXene/Ecoflex nanocomposite by using the 3D printing technology, as depicted in Fig. 11h [177]. 3D printing has emerged as a transformative approach to improving the wearability of triboelectric sensor, offering customized designs, lightweight materials, and flexible form factors tailored to individual users. Therefore, a 3D-printed toroidal structure was implemented along the whole glove portion using a flexible TPU filament for user convenient wear. With as-fabricated smart glove, they succeed the control of gaming car and balancing ball game on plate, which require complicated control ability. Fang et al. presented research that could open the horizons of smart wearable technologies by suggesting the passive response of triboelectric wearable sensors from wrist muscle/tendons, in Fig. 11i [178]. This sensor enable to identify sophisticated hand gestures with adaptive accelerated learning (AAL) with the advantages of low cost, high shape adaptability, and noninvasive wear based on the assistance of an adjustable strap for user. By developing an accelerated neural network for gesture recognition and enhancing its efficiency and reliability through an adaptive pruning model, we successfully integrated an optimized machine learning algorithm with intelligent wristbands. This system achieved 97.56% classification accuracy for 21 hand gestures, with an average accuracy of 95.41% across 7 participants. In summary, this section details the implementation of AI-assisted triboelectric wearable sensors in hands-free device control, emphasizing their advantages over traditional input methods and their potential applications in human–machine interaction.

### Metaverse

**Fig. 12:**
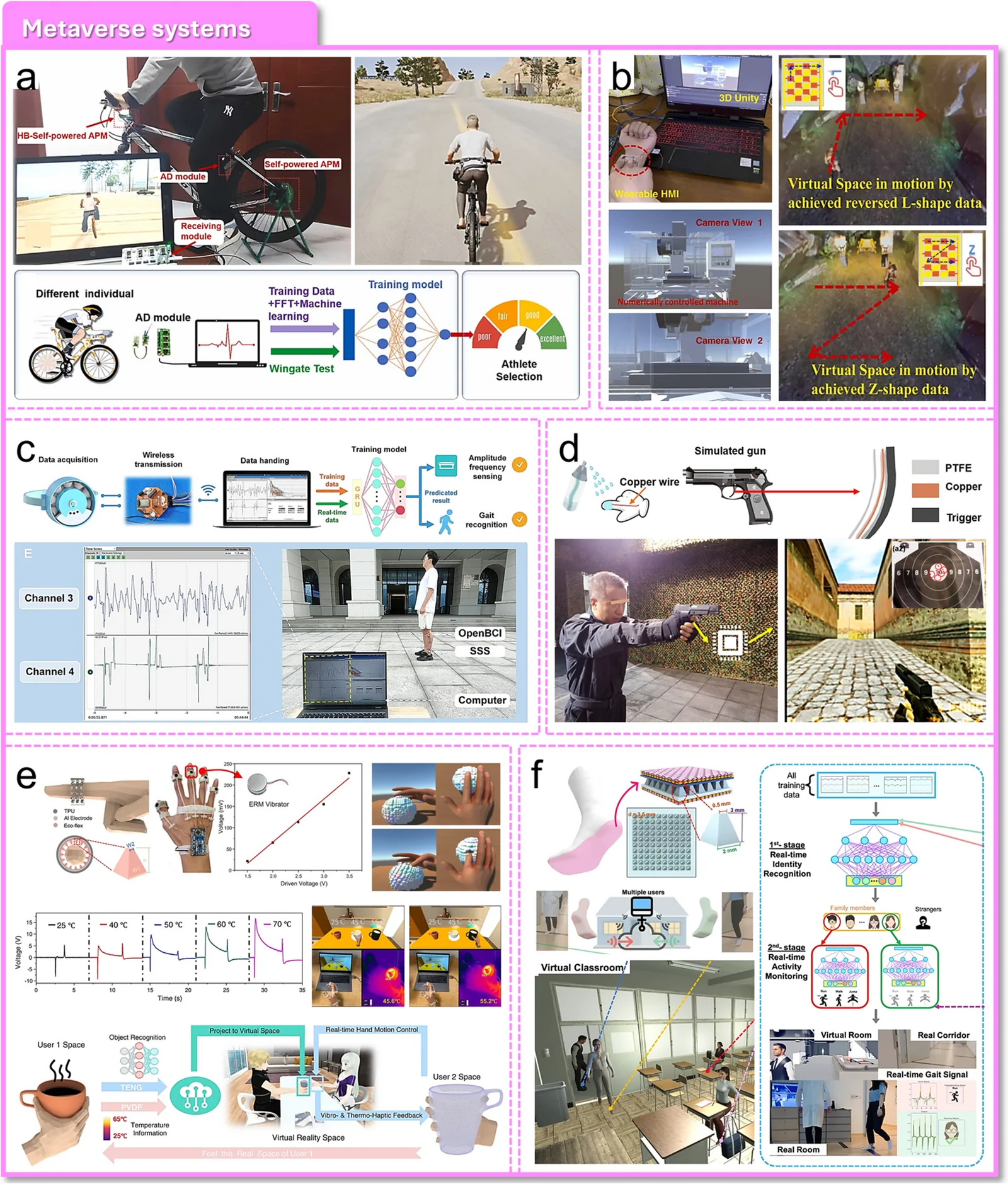
Applications of metaverse. **a** A real–virtual interaction system. Reprinted with permission from Ref. [182]. © 2023 Elsevier Ltd. All rights reserved. **b** A self-powered HMI sensor and virtual game activities applications. Reprinted with permission from Ref. [183]. © 2022 Elsevier Ltd. All rights reserved. **c** Amplitude–frequency sensing and gait recognition based on deep learning. Reprinted with permission from Ref. [182]. © 2024 Elsevier B.V. All rights are reserved, including those for text and data mining, AI training, and similar technologies. **d** A self-powered intelligent shooting system for improving police shooting technology. Reprinted with permission from Ref. [185]. Songyang Li et al., Materials, published by MDPI, 2022. **e** Multimodal sensing and feedback platform for metaverse. Reprinted with permission from Ref. [172]. Copyright © 2022, Zhongda Sun et al. **f** Multimodal sensing and feedback platform for metaverse. Reprinted with permission from Ref. [186]. Copyright © 2020, Zixuan Zhang et al.

The metaverse is an emerging digital ecosystem that relies on seamless interaction between users and virtual environments [179]. Conventional input devices such as controllers and motion trackers limit the immersive experience due to physical constraints and power consumption [180]. Triboelectric wearable sensors provide an innovative solution by enabling real-time motion tracking, ensuring uninterrupted engagement in virtual spaces [181]. AI algorithms process sensor data to translate physical movements into precise digital actions, improving avatar control, haptic feedback, and interaction realism. Zhu et al. proposed a metaverse sport interactive system, which is composed of a self-powered anaerobic power meter (APM) based on polyurethane (PU) and MXene/polytetrafluoroethylene (MXene/PTFE) as the friction layer [182]. By using Fourier discrete transforms, the speed of a bicycle with APM can be obtained, providing information and anaerobic power testing for the improvement of athletes, as presented in Fig. 12a. The machine learning system classifies sensing signals into four grades with a recognition accuracy of 95.37%. To enhance engagement in exercise, a real–virtual game interactive system is developed using IoT nodes for seamless interaction. In recent, metaverse technology has been attracting attention not only in sports/games but also in industry. Zhu et al. exhibited a sustainable machine learning-augmented dual-electrode TENG of a self-powered HMI sensor for motion detection and virtual reality [183]. They employed the principal component analysis (PCA) algorithm, t-distributed stochastic neighbor embedding (t-SNE) algorithm, Gaussian mixed model (GMM) algorithm, and K-means clustering algorithm, all machine learning-based methods, effectively enhanced identification accuracy by reinforcing distinctions between different datasets, improving motion pattern recognition and decoupling for more precise classification. Therefore, in Fig. 12b, the self-powered HMI sensor can recognize our hand motions and switch cameras angle or manipulate movements in the virtual world, presenting the potential for the development of digital twin technology in industrial settings. On the other hand, triboelectric wearable sensor can be used to collect user data for gathering personalized experiences and real-time interactions in the metaverse, driving its advancement. Yang et al. developed an innovative self-powered sensing smart monitoring system (SSSMS) that harvests low-frequency inertial energy from human motion, enabling gait recognition and fall monitoring (GR-FM) capabilities, as shown in Fig. 12c [184]. The electrical signals representing the conditions captured are transmitted to the computer terminal in real time via OpenBCI, and the GRU deep learning model analyzes and extracts features, which can not only accurately recognize different amplitudes and frequencies but also precisely recognize eight different gait patterns with recognition accuracy of 96.13%, 96.60%, and 92.22%, respectively. Finally provided a personalized and intelligent implementation interaction experience, demonstrating its potential as an interactive device for the next generation of metaverses. Additionally, the metaverse could also be an excellent training ground for police and military personnel to improve their shooting skills. Le et al. proposed a self-powered intelligent shooting system with electronic skin to monitor physiological and biochemical indexes including hand muscle motion track, force direction, and joint angle change, as presented in Fig. 12d [185]. In this study, a human–computer interaction system is used to collect and identify signals. SPTENG, the detection device of the human–computer interaction system, provides accurate detection signals and connects real shooting behavior with virtual games, offering military police with safe and valuable experience and training, which can be provided as a cheap and universal solution for shooting action and stability. In order for our characters' movements in the metaverse to be varied and active, we need to be able to capture the movements. Therefore, for user-friendliness, here are some research that can be applied to representative hands and feet. Sun et al. developed a augmented tactile perception and haptic feedback rings with multimodal sensing and mutual feedback capabilities [172]. The integrated units on the ring can be operated directly by a custom wireless platform considering the wearable/portable scenarios. With respect to the integration of both multimodal sensing and feedback function, it enables the users to be immersive in the interactive metaverse platform. In particular, they demonstrated a variety of virtual situations, such as grip object, item reconstruction, and collision detection, as shown in Fig. 12e. According to the prospect of the metaverse infrastructure, the AI-assisted fusion of multimodal somatosensory for full-body perception and feedback with VR display technology can build a smart virtual society capable of intelligent and interactive social, education, entertainment, and health care. Similarly, Zhang et al. reported a deep learning-enabled triboelectric smart socks capable of gathering comprehensive monitoring information of the user’s physical status in long-term in Fig. 12f [186]. In addition, the deep learning technology is employed for data analysis with the intelligent socks, realizing the gait identification of group users with the accuracy of 93.54%. Based on this high accuracy, the intelligent sock can be applied in the virtual reality fitness game, offering the possibility of a control interface with the shared vision and voice control terminal for augmented interactions. Consequently, this platform of intelligent smart sock contributes to the realization of the digital human technology and virtual world in near future. This section discusses the integration of triboelectric sensors in metaverse applications and their potential to revolutionize virtual reality (VR) and augmented reality (AR) interactions [187].

### Wearable-to-Robotic Extensions of AI-Assisted Triboelectric HMI

**Fig. 13:**
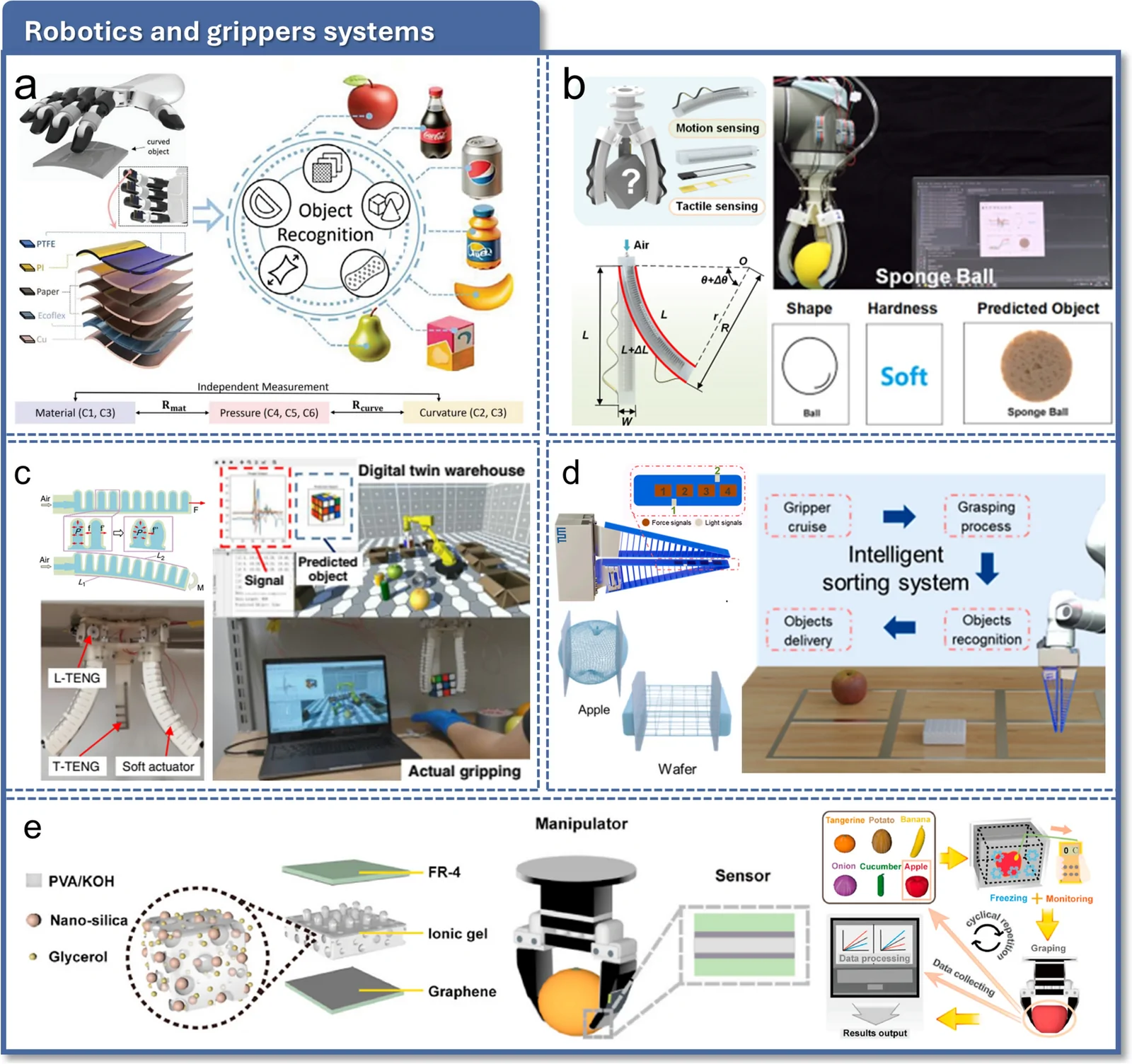
Applications of robotics and soft gripper. **a** Triboelectric multimodal tactile sensor (TMTS) with intelligent robotic finger for object recognition. Reprinted with permission from Ref. [10]. © 2024 Wiley‐VCH GmbH. **b** Soft gripper based on multimodal sensing for real-time recognition by using the trained CNN model. Reprinted with permission from Ref. [191]. Copyright © 2024, American Chemical Society. **c** Smart gripper integrated digital twin unmanned warehouse system. Reprinted with permission from Ref. [192]. Copyright © 2020, The Tao Jin et al. **d** Smart grasp by the tactile perception system for intelligent sorting system. Reprinted with permission from Ref. [193]. © 2024 Tianxiao Xiao et al., Published by Elsevier Ltd. **e** Object recognition function of smart gripper for grabbing different fruits and vegetables. Reprinted with permission from Ref. [194]. © 2024 Elsevier Ltd. All rights reserved

Beyond wearable applications, triboelectric wearable sensors are increasingly being integrated into robotic systems and soft grippers [188]. In robotics, precise sensing and adaptive control are essential for dexterous manipulation and object handling [189]. Triboelectric sensors enable robots to detect contact forces, grasp objects securely, and adjust their grip based on real-time feedback [190]. AI-assisted processing further enhances robotic perception, allowing for intelligent decision-making in complex environments. Zhao et al. presented a triboelectric multimodal tactile sensor (TMTS) designed to intelligently recognize material, curvature, and pressure simultaneously, as depicted in Fig. 13a [10]. In particular, it is noteworthy that the ability of quantitative curvature measurements provides deeper insights into an object’s shape. This enhanced precision allows for a more accurate differentiation between objects that share similar overall shapes but exhibit distinct curvatures. By integrating detected material properties, curvature, and pressure signals into a deep learning algorithm, it is established that a highly precise and robust object recognition system is capable of functioning across a wide range of applied forces. Furthermore, by analyzing the detected pressure and curvature data the TMTS system can be further refined to assess and identify an object’s softness, expanding its applicability in tactile sensing and material classification, providing a sense of touch sensory system to a robot hand. The accuracy of TMTS can be reached up to 94.1%, expanding the potential in a wide range of applications including the automated sorting systems and exploration in extreme environments. Moreover, grippers similar to humanoid robot hands are also ideal applications for AI-assisted triboelectric wearable sensor. Wang et al. exhibited that an intelligent soft robotic system with autonomous operation and multimodal perception capabilities can be replaced by integrating capacitive sensors with triboelectric sensors, in Fig. 13b [191]. Equipped with multiple distributed sensors, this gripper can not only sense and store multimodal information but also implement an adaptive grasping strategy for precise robotic positioning and grip control. The multimodal sensory data are captured with high sensitivity and fused at the feature level, enabling cross-modal object recognition. This integration significantly enhances the recognition capability, making it more efficient in dynamic and complex environments. In the other case of gripper-type soft robot, Jin et al. reported the potential of AI-assisted triboelectric wearable sensor for the soft gripper in the applications of digital twin [192]. A smart soft robotic gripper system is based on TENG to recognize the continuous motion and tactile information about various types of objects, as shown in Fig. 13c. Herein, the triboelectric sensory information is trained by support vector machine algorithm for recognition of diverse objects with high accuracy of 98.1%, enabling the object identification and duplication of robotic control in virtual environment in real time. Xiao et al. showed the different types of sensory system, which is a multidimensional tactile perception for smart grasp, as illustrated in Fig. 13d [193]. To have a higher sensitivity, a wavy structure is applied onto the surface of the PDMS composite film. Moreover, the condition-dependent luminescent effect is used by the functional addition of cadmium selenide/cadmium sulfide (CdSE/CdS) QRs. With this configuration, the eight triboelectric sensors-based tactile perception is realized by the integration of a soft gripper with a computer-controlled robotic arm toward intelligent sorting application capable of picking and placing for grasping and positioning object. Lastly, Liu et al. proposed a tri-modal tactile sensor with a porous ionic hydrogel in Fig. 13e [194]. Importantly, in order to maintain the performance, the pressure-sensitive and thermal-sensitive properties remain largely independent of each other due to ionic hydrogel network, which provides excellent water retention, antifreezing properties, and heat insulation capability. The multifunctional tactile sensor is integrated into a soft gripper to capture both force and thermal information of the measured object, and when combined with machine learning algorithms, enables accurate object recognition. This proposed sensing system has been successfully applied to the classification of six types of fruits and vegetables in a cold chain logistics scenario, achieving an impressive classification accuracy of 95%. Therefore, triboelectric wearable sensors, as demonstrated in this review, hold significant potential for applications in humans, humanoid robots, and even robotic grippers. Their real-time responsiveness makes them particularly valuable for prosthetic devices, where precise and immediate feedback is crucial. Furthermore, the integration of these sensors with AI brings the prospect of practical implementation closer to reality, paving the way for more intelligent and adaptive human–machine interfaces. This section explores how AI-assisted triboelectric sensors are redefining automation in industrial robotics, medical prosthetics, and autonomous systems.

### AI-Assisted Triboelectric Sensing for Intelligent Transportation and Advanced Mobility

**Fig. 14:**
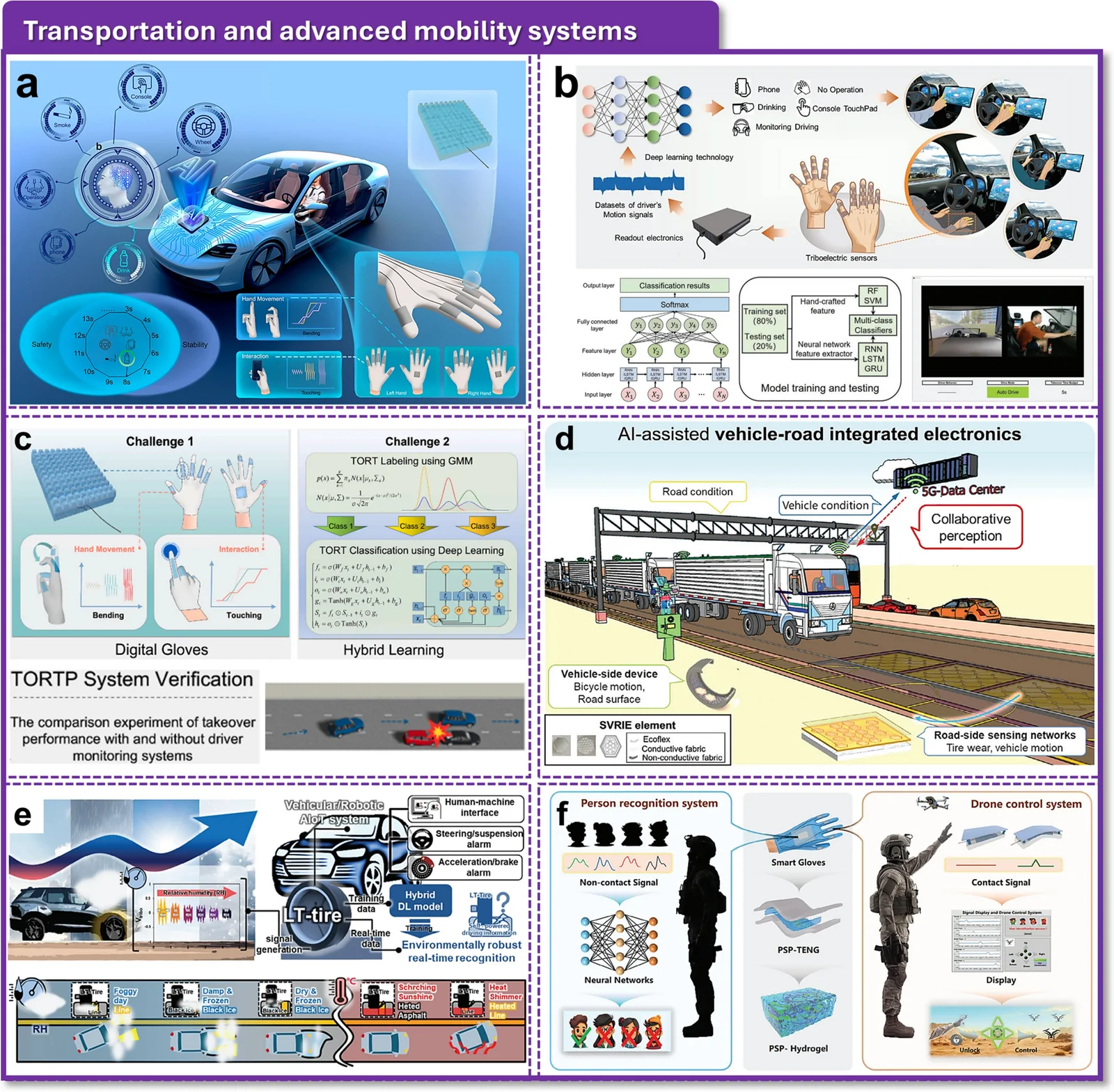
Applications of intelligent transportation and advanced mobility. **a** Wearable triboelectric sensor gloves for real-time behavior identification and takeover time adjustment in conditionally automated vehicles. Reprinted with permission from Ref. [195]. Copyright © 2025, Xiao Lu et al. **b** Real-time non-driving behavior recognition using deep learning-assisted triboelectric sensors in conditionally automated driving. Reprinted with permission from Ref. [196]. © 2022 Wiley‐VCH GmbH. **c** Inference of takeover time budget for level 3 autonomous vehicles using triboelectric sensors and hybrid learning. Reprinted with permission from Ref. [197]. © 2025 Elsevier B.V. All rights are reserved, including those for text and data mining, AI training, and similar technologies. **d** AI-assisted self-powered vehicle–road integrated electronics for intelligent transportation collaborative perception. Reprinted with permission from Ref. [198]. © 2024 Wiley‐VCH GmbH. **e** Environmentally robust triboelectric tire monitoring system for self-powered driving information recognition. Reprinted with permission from Ref. [199]. © 2024 Wiley‐VCH GmbH **f** Self-healing hydrogel-based triboelectric smart glove for integrated drone safety protection and motion control. Reprinted with permission from Ref. [200]. © 2024 Wiley‐VCH GmbH

Beyond conventional healthcare and gesture recognition scenarios, AI-assisted triboelectric sensing is increasingly being extended to intelligent transportation and advanced mobility, where real-time state awareness, adaptive decision-making, and intuitive control are critically required. In this regard, Fig. 14 summarizes a coherent technological progression from wearable takeover assistance interfaces in automated vehicles, to self-powered road-aware sensing systems, and finally to advanced mobility control platforms enabled by triboelectric HMIs. First, in Fig. 14a, Lu et al. reported all-round sensing gloves based on triboelectric sensors seamlessly detected delicate hand motions and hand–object interactions in the vehicle cabin, and the acquired signals were processed by a non-driving behavior identification module to classify six representative non-driving behaviors with an accuracy of 94.72% [195]. The identified behavior was then linked to a takeover time budget determination module, enabling dynamic adjustment of the takeover time budget according to the driver’s current non-driving state and significantly improving takeover safety and stability in L3 automated driving. This study clearly demonstrates that triboelectric glove interfaces can serve as active in-cabin safety modules for human-centered automated vehicle interaction. Additionally, Zhang et al. presented real-time non-driving behavior recognition using deep learning-assisted triboelectric sensors in conditionally automated driving (RNBRS), as illustrated in Fig. 14b [196]. In contrast to fixed takeover warning strategies, this study proposed a real-time non-driving behavior recognition system integrating self-powered triboelectric sensors mounted on the hand and a deep learning-assisted long short-term memory network. The system recognized five representative non-driving behaviors, including phone use, console touchpad interaction, driving, monitoring driving, and no operation, with a test accuracy of 93.5%. On the basis of the recognition result, the takeover time budget could be dynamically adjusted according to the driver’s current behavior, thereby significantly improving both the safety and stability of vehicle takeover. This work established an important early example of triboelectric hand sensing for intelligent takeover prevention. Zhang et al. further proposed this direction in inference of takeover time budget for level 3 autonomous vehicles using triboelectric sensors and hybrid learning, as presented in Fig. 14c [197]. Rather than only recognizing non-driving behaviors, this study introduced the Takeover Recovery Time Inference system (TORTI), which integrated digital gloves with hybrid unsupervised and supervised learning to establish an end-to-end relationship between glove-derived signals and takeover recovery time. The digital gloves captured driver hand movements and interactions with various objects in the car in real time, and the proposed framework inferred takeover recovery time with an accuracy of 90.3%. By converting this inferred recovery requirement into a dynamically calibrated takeover time budget, the system significantly enhanced the safety, stability, and consistency of takeover in L3 autonomous vehicles. Compared with behavior-only recognition approaches, this work moved triboelectric glove sensing closer to direct, decision-relevant mobility intelligence. Beyond in-cabin takeover assistance, in Fig. 14d, Pang et al. proposed the same triboelectric sensing principle was implemented in both vehicle-side and roadside modules through self-powered vehicle–road integrated electronics, enabling collaborative perception of road conditions, vehicle motion, and tire life cycle health [198]. The proposed system achieved at least 88.3% accuracy for road condition recognition, 100% accuracy for transfer learning-assisted road surface identification, 97% accuracy for tire motion recognition, and 99% accuracy for tire health monitoring. Although this approach extends beyond strictly wearable sensing, it is highly relevant to the present review because it demonstrates how triboelectric sensing can expand from human-attached interfaces toward broader intelligent transportation ecosystems integrating vehicles and infrastructure. Similarly, as shown in Fig. 14e, Kim et al. reported a laser-induced graphene-based triboelectric tire monitoring system was designed to extract road contact information from tire–road friction signals, while short-time Fourier transform and a CNN–LSTM hybrid model were used to decouple driving information from environmental noise [199]. The system enabled recognition of driving information such as traffic lines and road states even under severe environmental fluctuations, including relative humidity from 10 to 90% and temperatures from 50 to 70 °C, and its real-time operation was verified on a mobile platform under foggy, damp, intense sunlight, and heat shimmer conditions. This work underscores that triboelectric sensing can provide a robust self-powered route toward road-aware mobility intelligence, especially when conventional vision-based perception becomes unreliable.

Importantly, the applicability of triboelectric sensing is not confined to ground transportation. As shown in Fig. 14f, Wang et al. demonstrated a smart glove based on a self-healing hydrogel triboelectric nanogenerator integrated both person recognition and gesture-based drone control into a single wearable platform [200]. Through non-contact sensing, personalized fingertip trajectories were recognized with an accuracy of 99.0%, enabling secure unlocking of the drone–control interface. Subsequently, contact sensing at the finger joints was used to convert hand gestures into precise drone flight commands. This study shows that the applicability of triboelectric glove interfaces is not confined to ground transportation, but can be further extended to advanced aerial mobility as a secure and intuitive HMI. Given that Fig. 14 indicates that AI-assisted triboelectric sensing is evolving along a clear mobility-oriented trajectory. It begins with wearable glove interfaces for intelligent takeover prevention in automated vehicles, expands toward self-powered perception of road, tire, and vehicle conditions, and ultimately extends to intuitive control of advanced mobility platforms such as drones. This progression highlights the broad potential of triboelectric sensing as a human-centered technological framework for future intelligent transportation and advanced mobility systems [201, 202]. To further summarize the representative application domains discussed above, Table 1 compares the major AI strategies, key strengths, and priority development needs of AI-assisted triboelectric wearable sensors toward practical use [203–205].

**Table 1:** Application domains, AI strategies, and priority development needs of AI-assisted triboelectric wearable sensors

Application domain	Used AI	Key strengths	Priority development needs	References
Health care	CNN, SVM, deep learning-based classification, patient-specific pattern analysis	Self-powered continuous monitoring of physiological and motion-related signals, strong potential for personalized and long-term health care	Clinical validation, long-term skin compatibility, reduction of motion artifacts, and secure data handling	[157, 159, 163, 165, 167, 168]
Gesture recognition and interactive input	CNN, machine learning, adaptive accelerated learning, multimodal signal classification	Direct detection of subtle hand and finger motions, privacy-friendly sensing, and intuitive human–input interfaces	Improved robustness across users, lower calibration burden, higher durability under repeated wear, and reduced latency	[29, 160, 170–172, 176]
Device control and smart interfaces	LDA, CNN, GRU, machine learning-assisted command recognition	User-adaptive control of electronic devices, robots, mobility systems, and assistive interfaces through self-powered sensing	Stronger interoperability with external devices, more reliable closed-loop operation, and improved command stability in real-world environments	[160, 161, 163, 170, 172, 175–178]
Metaverse and immersive virtual interaction	Deep learning, PCA, t-SNE, GMM, k-means, gait/motion recognition models	Real-time motion tracking, multimodal sensing and feedback, and enhanced immersion in virtual environments	Better synchronization between sensing and feedback, full-body multimodal integration, and stable long-term wearability	[172, 182, 183, 185, 186]
Robotics and intelligent manipulation	CNN, SVM, deep learning-based object recognition, multimodal feature fusion	Multimodal tactile perception, object recognition, adaptive grasping, and enhanced robotic interaction	Higher durability under repeated operation, denser multimodal integration, and more reliable real-time feedback for closed-loop control	[10, 191–194]
Intelligent transportation and advanced mobility	LSTM, TENN–LSTM, hybrid learning, STFT–CNN–LSTM	Driver-state-aware sensing, self-powered road and vehicle perception, and extension toward advanced mobility interfaces	Harsh environment reliability, larger-scale field validation, and safer integration with mobility platforms	[195–200]

### Representative Future Directions of AI-Assisted Triboelectric Wearable Sensors

**Fig. 15:**
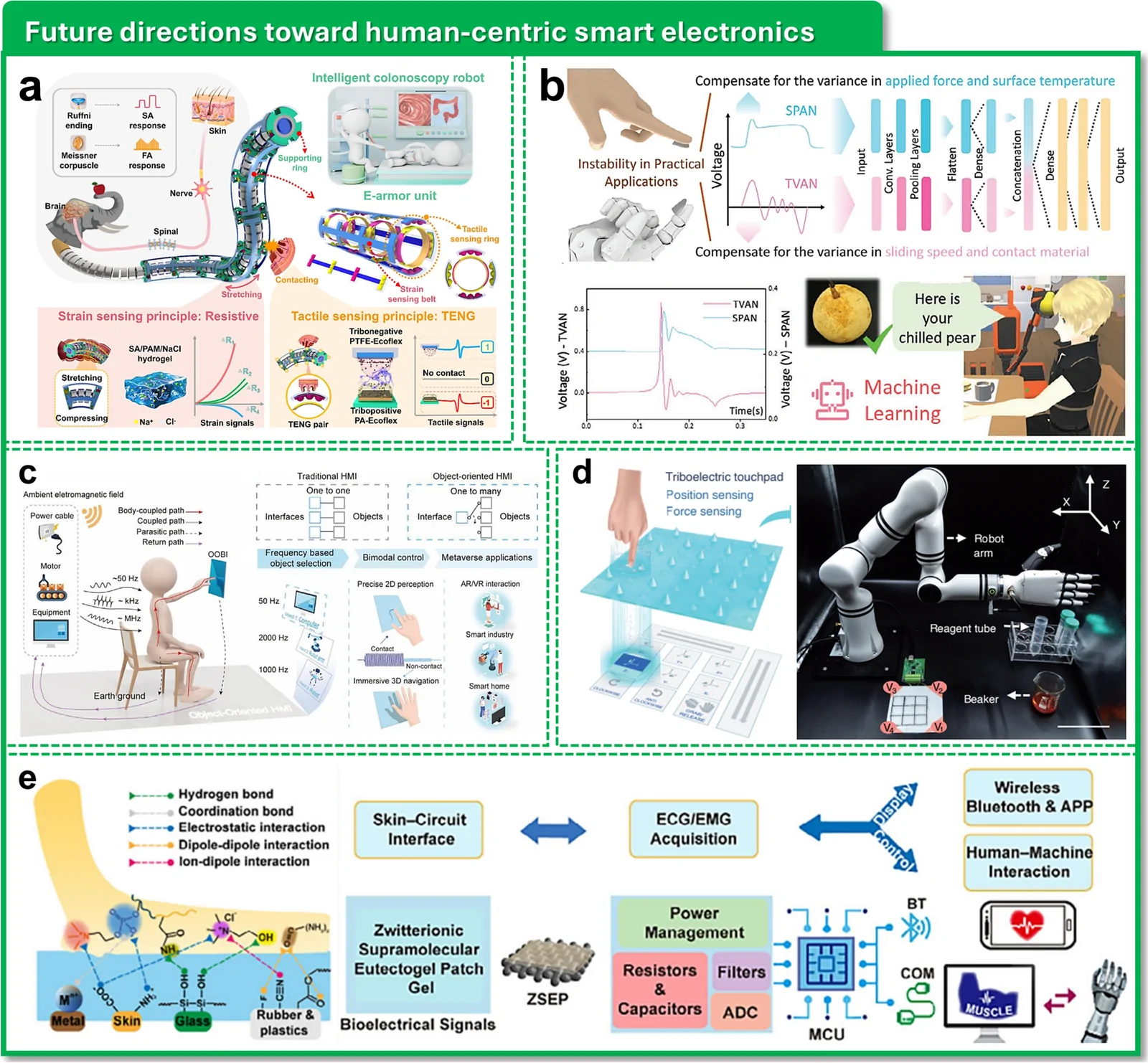
Future directions toward human-centric smart electronics. **a** Intelligent colonoscopy robot with stretchable electronic armor for bimodal tactile and strain sensing. Reprinted with permission from Ref. [207]. © 2025 Sun et al., Advanced Materials published by Wiley‐VCH GmbH. **b** Zero-biased bionic fingertip e-skin for multimodal tactile perception and machine learning-assisted holistic touch awareness. Reprinted with permission from Ref. [149]. © 2024 Wiley‐VCH GmbH. **c** Body-coupled-driven object-oriented natural interactive interface for hybrid 2D tactile and 3D spatial interaction. Reprinted with permission from Ref. [208]. © 2025 Wiley‐VCH GmbH. **d** Biomimetic hairy triboelectric touchpad for multimodal position and force sensing with dexterous robotic manipulation. Reprinted with permission from Ref. [150]. Copyright © 2026, Hong et al. **e** Skin-inspired supramolecular zwitterionic eutectogel interfaces for wearable sensing, physiological monitoring, and human–machine interaction. Reprinted with permission from Ref. [209]. © 2025 Wiley‐VCH GmbH

Looking ahead, recent advances suggest that AI-assisted triboelectric wearable sensors are evolving beyond conventional self-powered sensing platforms toward integrated intelligent systems capable of perception, interpretation, interaction, and adaptive response [206]. As summarized in Fig. 15, several representative future directions are emerging, spanning embodied medical robotics, holistic tactile cognition, spatially aware natural interfaces, affective human–AI communication, and skin-inspired iontronic material platforms.

First, Sun et al. developed a stretchable, multiplexed, and bimodal sensing electronic armor for a colonoscopic continuum robot, representing a future direction toward embodied medical intelligence, as described in Fig. 15a [207]. Their triboelectric artificial synapse-based e-armor enabled 300 mm full coverage with 48 tactile sensing points and 12 strain sensing edges, while a CNN–LSTM framework achieved 99.31% accuracy for multipoint tactile signal identification. By integrating tactile sensing, hydrogel-based strain sensing, compliance control, and digital twin-based posture visualization, this work indicates that triboelectric wearable systems may evolve into intelligent medical robotic interfaces capable of safe intervention, autonomous navigation, and real-time surgical feedback. Next, Guo et al. reported a zero-biased bionic fingertip e-skin with multimodal tactile perception and artificial intelligence, highlighting a future direction toward holistic tactile cognition, as presented in Fig. 15b [149]. By integrating transient voltage artificial neurons and sustained potential artificial neurons into a single-unit platform, the e-skin could simultaneously perceive vibration, material, texture, pressure, and temperature. Combined with feature fusion and machine learning, the system achieved 91.2% accuracy for roughness perception, 99.5% for hardness discrimination, 98.45% for object recognition under temperature variation, and 98.85% for temperature decoding. This study suggests that triboelectric wearable sensors may move beyond simple event detection toward comprehensive tactile intelligence for robotic fingertips, prosthetics, and embodied AI systems. Furthermore, Hong et al. proposed a body-coupled-driven object-oriented natural interactive interface, pointing to a future direction of spatially aware natural interfaces in Fig. 15c [208]. Their transparent and stretchable interface combined micrometer-scale 2D tactile precision with 3D spatial perception through body-coupled electromagnetic coupling, achieving 200 μm trajectory reconstruction precision, a 200 mm non-contact sensing range, and 97.11% recognition accuracy for 38 gestures. More importantly, they introduced an object-oriented human–machine interaction framework in which a single interface could select and control multiple devices through electromagnetic signature recognition. This work shows that future triboelectric wearable systems may expand toward universal spatial interfaces for metaverse interaction, multimachine collaboration, and natural cross-domain control. Beyond functional perception, Hong et al. further demonstrated a biomimetic hairy affective touch sensory AI interface, revealing a future direction toward affective human–AI communication, as demonstrated in Fig. 15d [150]. Inspired by hairy skin biomechanics and C-LTMR-like affective touch transduction, the system generated neuromimetic spikelike outputs without an external spike-coding circuit and captured multidimensional tactile information including force, position, and stroking speed. Through convolutional neural networks and large language models, it achieved 82.37% emotion recognition accuracy and established a closed-loop human–AI emotional interaction framework. This study expands triboelectric wearable sensing from physical interaction into emotional and social communication, suggesting future applications in empathetic robotics, mental health support, and companion systems. Finally, Zhou et al. presented initiator-free supramolecular zwitterionic gels for skin-inspired soft iontronics, emphasizing a future direction centered on iontronic material platforms in Fig. 15e [209]. Although this work is more materials-platform-oriented than device-specific, it is highly relevant because it provides the soft, biocompatible, and conductive foundation needed for next-generation wearable interfaces. The zwitterionic supramolecular eutectogel (ZSE) exhibited tunable mechanical softness, ionic conductivity up to 2 S m^−1^, recyclability, self-adhesion, antibacterial activity, and biocompatibility. It could be continuously spun into wearable filaments for motion and temperature sensing, while gel patches enabled physiological monitoring such as ECG and EMG as well as robotic hand control. This study suggests that the future development of triboelectric wearable sensors will depend not only on sensing functions, but also on robust, skin-compatible, and sustainable iontronic materials.

Hence, these studies indicate that the future of AI-assisted triboelectric wearable sensors will likely be shaped by the convergence of multifunctional perception, embodied intelligence, natural interaction, emotional communication, and bioinspired material platforms. Rather than remaining as self-powered sensing units alone, triboelectric wearable systems are expected to evolve into integrated intelligent interfaces that more directly connect humans, machines, and adaptive environments.

## Summary and Outlook

Triboelectric wearable sensors, a critical component of next-generation HMIs, have emerged as a self-powered solution for continuous and real-time sensing applications. Unlike conventional sensors that require external power sources, triboelectric sensors convert mechanical motion to generate electrical signals, making them ideal for integration into wearable and flexible electronics. With the integration of AI, these sensors have advanced beyond basic stimulus detection by enabling more sophisticated signal interpretation, adaptive analysis, and real-time decision-making. In this regard, AI primarily enhances how triboelectric signals are processed and utilized, whereas the intrinsic sensing characteristics remain governed by the materials, structures, and operating principles of the devices. Triboelectric sensors operate based on the triboelectric effect, where contact between two different materials induces charge transfer. This charge generation mechanism is influenced by material properties, surface structures, and fabrication techniques. The core components of triboelectric sensors include a triboelectric layer for charge transfer and an electrode layer for charge collection. Common materials for triboelectric layers include fluorinated polymers such as PTFE and FEP, natural materials such as paper and animal fur, and advanced composites like metal–organic frameworks and liquid-based interfaces. The fabrication of triboelectric sensors has evolved through various techniques, such as self-assembly, electrospinning, and additive manufacturing, enabling the development of flexible, lightweight, and biocompatible sensors. These sensors are designed with multiple operational modes, including vertical contact–separation, lateral sliding, single-electrode, and freestanding modes, each optimized for specific applications. The continuous improvements in triboelectric material engineering and fabrication strategies have expanded the functionality and performance of triboelectric wearable sensors.

**Fig. 16:**
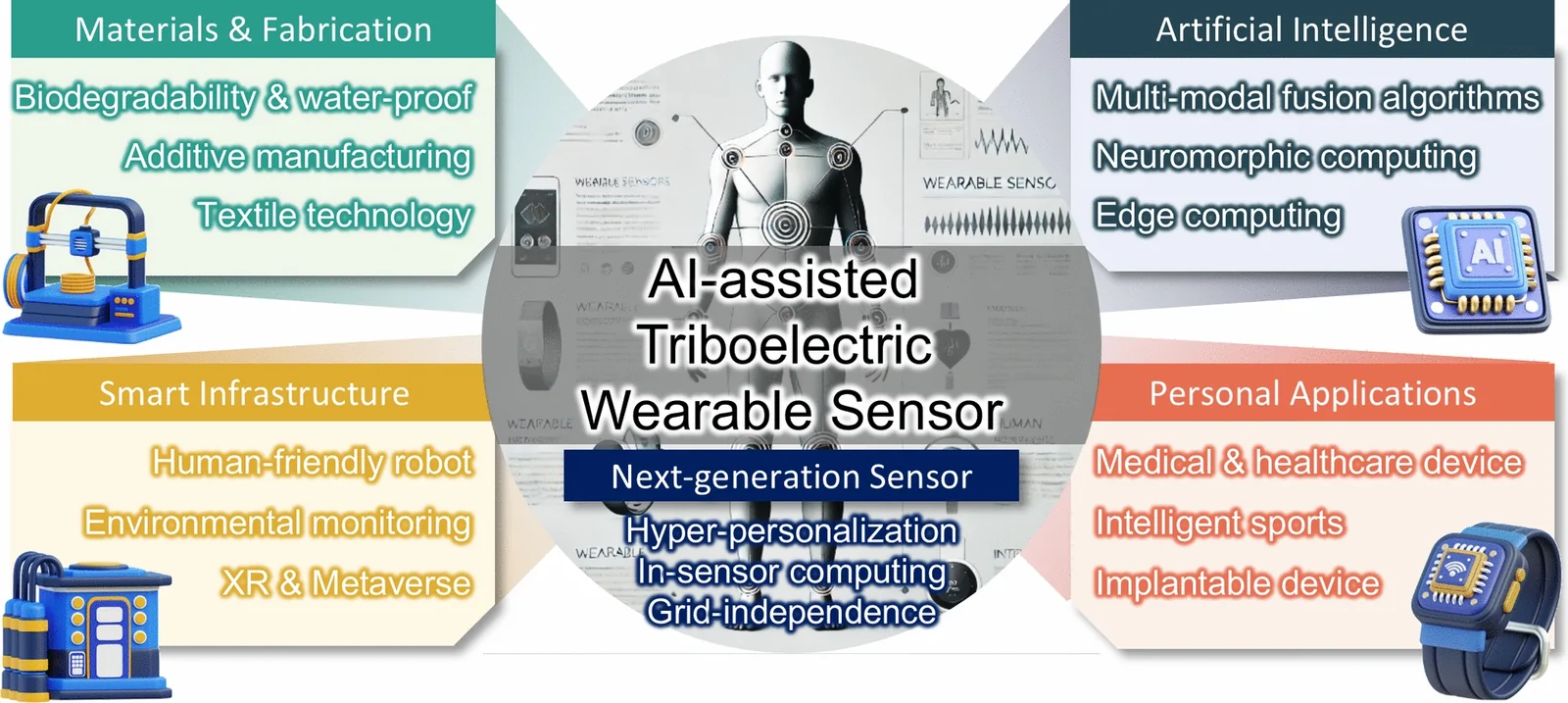
Summary, challenges, and perspectives of AI-assisted triboelectric wearable sensor

The integration of AI with triboelectric sensors has significantly enhanced their capabilities, enabling real-time data processing, pattern recognition, and predictive analytics. AI-driven signal processing techniques, including deep learning and neuromorphic computing, improve the analysis efficiency and inference performance of triboelectric sensing systems by extracting discriminative features, suppressing noise, and enabling more robust classification and prediction. AI models, such as convolutional neural networks (CNNs) and recurrent neural networks (RNNs), refine the interpretation of sensor output**s** by filtering noise, extracting task-relevant temporal and spatial features, enabling adaptive learning, and improving classification accuracy under variable real-world conditions. The development of neuromorphic computing architectures mimics biological synapses, facilitating intelligent sensory data interpretation. Additionally, AI-assisted triboelectric sensing systems enable more effective multimodal interpretation of mechanical, thermal, and electrical inputs when these signals are acquired simultaneously, thereby improving data integration and contextual understanding rather than directly changing the intrinsic transduction sensitivity of the sensor itself. The integration of AI with triboelectric sensors has unlocked new possibilities for advanced HMI applications, including gesture recognition, tactile sensing, and autonomous decision-making in robotics. The provided illustration visually summarizes the role of AI in triboelectric wearable sensors, showcasing their applications across various domains in Fig. 14. Key elements include material advancements such as biodegradable, waterproof, and additive-manufactured sensors, AI-driven enhancements like multimodal fusion algorithms and neuromorphic computing, smart infrastructure including environmental monitoring, robotics, and metaverse applications, and personal applications such as medical devices, intelligent sports equipment, and implantable sensors. Figure 16 emphasizes how AI augments the efficiency and adaptability of triboelectric sensors, enabling seamless HMIs.

The integration of AI-assisted triboelectric wearable sensors has shown strong potential across multiple fields, particularly in improving user experience, accessibility, and interactivity. However, at the present stage, most of these systems remain largely within laboratory validation and prototype-level demonstration, and further efforts are still required for large-scale implementation, long-term reliability, and practical deployment. AI-assisted triboelectric wearable sensors enable continuous health monitoring, tracking physiological signals such as heart rate, respiratory patterns, and rehabilitation progress. The incorporation of machine learning algorithms allows for early disease detection and personalized treatment plans. Triboelectric sensors facilitate real-time gesture-based control, improving accessibility in assistive technologies, gaming, and augmented reality. AI enhances gesture classification accuracy, ensuring seamless user interaction. These sensors provide an intuitive interface for controlling electronic devices, smart home systems, and industrial automation tools. AI-driven learning algorithms optimize response accuracy and user-specific adaptability. AI-assisted triboelectric sensors enhance VR and AR experiences by enabling precise motion tracking and haptic feedback. These sensors improve avatar control, object interaction, and real-time engagement in digital environments. In robotic applications, triboelectric sensors enhance object recognition, force detection, and adaptive grasping [210]. AI-driven perception systems enable soft grippers to manipulate objects with precision, expanding their use in industrial automation and prosthetics. Figure 16 illustrates these diverse applications, highlighting the AI-enhanced capabilities of triboelectric wearable sensors in health care, gesture recognition, device control, the metaverse, and robotics. The image visually demonstrates the integration of AI and triboelectric sensors to improve interaction quality and system efficiency.

Despite their promising potential, triboelectric wearable sensors face several challenges that need to be addressed for broader commercialization and real-world adoption. For applications in medical and implantable devices, triboelectric sensors have to exhibit high biocompatibility and long-term stability without causing adverse biological reactions [211]. To reduce latency and power consumption, future research should focus on integrating AI-driven computing within the sensor itself, enabling real-time processing without reliance on external computation units [53]. Enhancing the energy harvesting efficiency of triboelectric sensors will enable them to function independently from traditional power sources, supporting sustainable and off-grid applications. Scalable production methods, including 3D printing and roll-to-roll fabrication, are essential to reducing manufacturing costs and making triboelectric sensors commercially viable. By addressing these challenges, triboelectric wearable sensors can fully realize their potential in next-generation smart electronics, paving the way for advanced HMI solutions across various industries. The continuous evolution of AI-integrated triboelectric sensors will redefine the future of seamless human–machine interactions, enhancing the efficiency, adaptability, and sustainability of intelligent systems.
